# Ambient outdoor heat and accelerated epigenetic aging among older adults in the US

**DOI:** 10.1126/sciadv.adr0616

**Published:** 2025-02-26

**Authors:** Eun Young Choi, Jennifer A. Ailshire

**Affiliations:** Leonard Davis School of Gerontology, University of Southern California, McClintock Avenue, CA90089, Los Angeles, CA 3715, USA.

## Abstract

Extreme heat is well-documented to adversely affect health and mortality, but its link to biological aging—a precursor of the morbidity and mortality process—remains unclear. This study examines the association between ambient outdoor heat and epigenetic aging in a nationally representative sample of US adults aged 56+ (*N* = 3686). The number of heat days in neighborhoods is calculated using the heat index, covering time windows from the day of blood collection to 6 years prior. Multilevel regression models are used to predict PCPhenoAge acceleration, PCGrimAge acceleration, and DunedinPACE. More heat days over short- and mid-term windows are associated with increased PCPhenoAge acceleration (e.g., *B*_prior7-dayCaution+heat_: 1.07 years). Longer-term heat is associated with all clocks (e.g., *B*_prior1-yearExtremecaution+heat_: 2.48 years for PCPhenoAge, *B*_prior1-yearExtremecaution+heat_: 1.09 year for PCGrimAge, and *B*_prior6-yearExtremecaution+heat_: 0.05 years for DunedinPACE). Subgroup analyses show no strong evidence for increased vulnerability by sociodemographic factors. These findings provide insights into the biological underpinnings linking heat to aging-related morbidity and mortality risks.

## Teaser

Ambient outdoor heat, especially long term, may accelerate epigenetic aging in a diverse national cohort of older adults.

## INTRODUCTION

Global warming has intensified extreme heat events, posing serious risks to public health (*1*). The frequency, intensity, and duration of extreme heat events are expected to grow rapidly in the coming decades, affecting more than 100 million Americans in 2050 (*2*). Extreme heat contributes to a range of health conditions, including hospitalization (*3*), cardiovascular diseases (*4*), and death (*5*). Health impacts from heat are particularly adverse among older adults due to age-related declines in thermoregulatory functions (*6*). Although links between extreme heat and morbidity and mortality are well established, knowledge of the biological underpinnings is limited.

The physiological toll exacted by heat events may not manifest immediately as clinical conditions. Rather, these environmental insults may elicit subclinical deterioration at the biological level, accelerating biological aging, which precedes the subsequent development of diseases and disabilities (*7*). Animal studies suggest that epigenetic alteration is a strong candidate for a potential biological mechanism (*8*, *9*). Severe heat stress can induce a “maladaptive epigenetic memory,” which can be coded through changes in DNA methylation (DNAm) patterns. DNAm, arguably the most well-studied epigenetic marker, is known to be responsive to environmental stressors, modulating gene expression and exerting downstream effects on morbidity and mortality risks (*10*). The effects of heat on DNAm have been documented across various species, including *Caenorhabditis elegans* (*11*), fish (*9*), and chickens (*12*), as well as mammals such as guinea pigs (*13*) and mice (*14*, *15*). Notably, a single episode of extreme heat stress can lead to long-lasting DNAm changes in mice, affecting cellular signaling pathways across tissue types, such as immune cells (*14*) and ventricular cardiomyocytes (*15*). While this body of work suggests that heat can alter the DNA methylome, with potential phenotypic changes (e.g., disease onset), very few studies have been done in humans (*16*, *17*).

Our study bridges this gap by examining epigenetic age (or clock), a molecular marker of biological aging based on DNAm levels throughout the genome (*18*). To our knowledge, only two studies (*19*, *20*) have investigated the relationship between heat and epigenetic age. Ni *et al.* (*19*) analyzed 1725 and 1877 participants from the Cooperative Health Research in the Region of Augsburg (KORA) F4 and follow-up FF4 cohorts in Augsburg, Germany and found that high temperature, defined as the 8-week average air temperature at 97.5th percentile (18.7°C) compared to the median temperature (9.7°C), was associated with increased epigenetic age acceleration, with a range between 1.83 and 11.71 years across different clocks. In addition, a 1°C increase in annual average temperature was related to epigenetic age acceleration, ranging from 0.24 to 2.24 years. Another study by Chiu *et al.* (*20*) analyzed 2084 cancer-free participants aged 30 to 70 from the Taiwan Biobank and showed that each unit (degrees Celsius) increase in the 60- and 90-day average air temperature was associated with epigenetic age acceleration by up to 44.3 days. However, these studies were restricted to specific samples from a single study center or had certain criteria for enrollment, which limits the generalizability of the findings across diverse populations with different racial, ethnic, and geographic backgrounds. Thus, there remain considerable limitations in our understanding of the broader population-level epigenetic aging implications of heat.

**Fig. 1.**
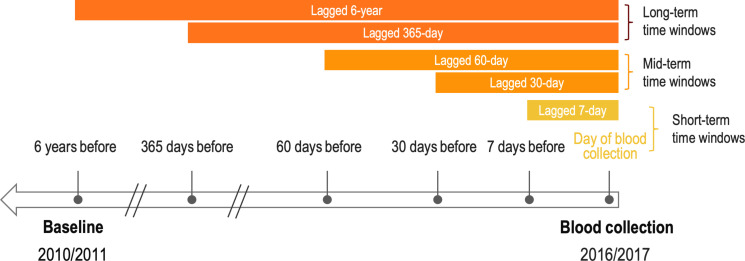
Ambient outdoor heat measures across time windows.

The goal of the present study is to assess the role of ambient outdoor heat in epigenetic aging by leveraging data from a nationally representative sample of US older adults. We examine multiple time windows, ranging from the day of blood collection (BC day) to a prior 6-year period, to determine whether epigenetic aging is responsive to both acute and chronic heat in the outdoor residential environment (Fig. 1). Furthermore, we explore sociodemographic differences (age, gender, race/ethnicity, education, and wealth) in these associations.

## RESULTS

### Sample characteristics

Table 1 shows the weighted sample characteristics. The mean chronological age of participants is 68.6 years (SD = 9.2 years), and 54% are women. The racial composition is predominantly non-Hispanic white (78%), followed by non-Hispanic Black (10%), Hispanic (8.6%), and other non-Hispanic races (3.4%). Participants have an average of 13.3 years of education, and the mean household wealth is $169,670. In terms of health behaviors, 35.7% are obese, 11% are current smokers, and 43.7% are current alcohol consumers, with 37% being light drinkers and 6.6% heavy drinkers. 61.8% report engaging in sufficient physical activity, defined as moderate activity more than once a week or vigorous activity at least once a week. The average tract level social vulnerability score is 0.48. 49.9% of participants live in urban areas, 24% in suburban areas, and 26.1% in ex-urban areas.

**Table 1.** Descriptive statistics for study sample, 2016 health and retirement study venous blood study DNAm subsample (*N* = 3686). Estimates are weighted using the survey weights.

Variables	*M* ± SD	Range (min–max)	%
*Sociodemographic and health behavioral profiles*			
Age (unit: years)	68.6 ± 9.22	56–100	
Women			54.0%
Race/ethnicity			
Non-Hispanic white			78.0%
Non-Hispanic Black			10.0%
Hispanic			8.6%
Non-Hispanic Other *			3.4%
Education (unit: years)	13.3 ± 2.97	0–17	
Non-housing household financial wealth (unit: $)	169,670 ± 716,867	−1800K–19,800K	
Smoking			
Never			44.4%
Former			44.6%
Current			11.0%
Drinking			
Noncurrent drinker			56.3%
Light drinker			37.0%
Heavy drinker (7 days a week)			6.6%
Obesity (yes)			35.7%
Sufficient physical activity (yes)			61.8%
Social vulnerability index^†^ (unit: percentile ranking)	0.48 ± 0.29	0–1	
Urbanicity			
Urban			49.9%
Sub-urban			24.0%
Ex-urban			26.1%
*Epigenetic aging* ^‡^			
PCPhenoAge Acceleration (unit: years)	−0.44 ± 5.58	−21.95–43.02	
PCGrimAge Acceleration (unit: years)	−0.23 ± 3.30	−9.72–14.06	
DunedinPACE (unit: rate of aging per year)	1.02 ± 0.15	0.63–1.67	

*Non-Hispanic Other group includes individuals identifying as American Indian, Alaskan Native, Asian, and Pacific Islander.

†Each census tract is assigned a percentile rank relative to all other tracts across the US, with higher values representing greater vulnerability.

‡For age acceleration measures, values represent the deviation of epigenetic age from chronological age, measured in years. Positive values indicate accelerated biological aging, while negative values indicate decelerated aging. For DunedinPACE, the unit represents the rate of estimated biological aging per year, with a value of 1 indicating a normal pace, where epigenetic age increases by 1 year for each chronological year.

Table 1 also presents the descriptive characteristics of the epigenetic aging measures. PCPhenoAge and PCGrimAge acceleration have means of −0.44 and −0.23 years, suggesting slower epigenetic aging on average in this sample. In contrast, the DunedinPACE, representing the pace of physiological decline per year, has a mean value of 1.02, suggesting that, on average, the sample is aging biologically 2% faster than the calendar year rate.

### Ambient outdoor heat distribution

**Fig. 2.**
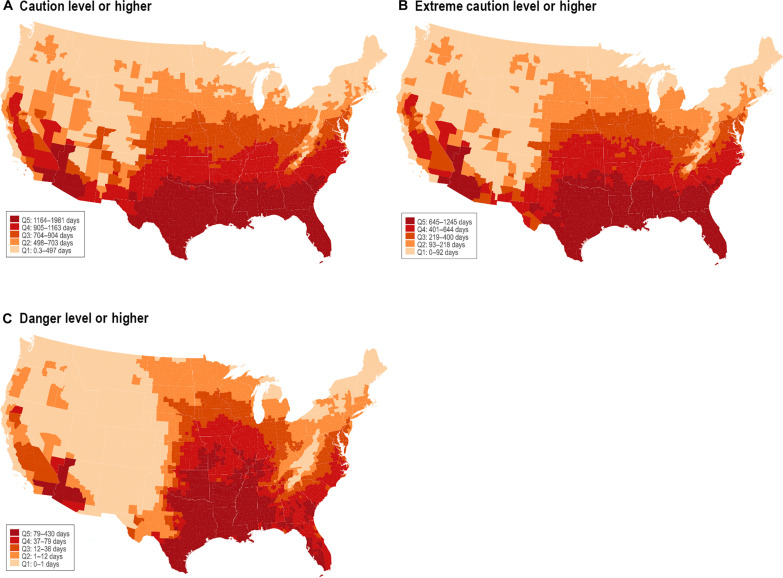
Total number of heat days over 2010–2016 across the Contiguous US. Heat days are defined as days on which the daily maximum heat index (HI) meets the thresholds provided by the National Weather Service (NWS) heat index chart, which categorizes HI values based on the potential risk of adverse health effects. The caution level includes HI values ranging from 80° to 90°F (26.7° to 32.2°C). The extreme caution level includes values between 90° and 103°F (32.2° and 39.4°C). The danger level encompasses HI values between 103° and 124°F (39.4° and 51.1°C). The total number of heat days meeting or exceeding each heat category is computed for each census tract for the period from 2010 to 2016 years. Country-level averages are then calculated by averaging tract-level data within the country. Counties are categorized in quintiles based on the total number of heat days, with the fifth quintile (Q5) representing counties with the highest number of heat days. (**A**) Total number of heat days at caution or higher levels. (**B**) Extreme caution or higher levels. (**C**) Danger or higher levels.

Figure 2 depicts the geographic distribution of heat days at different levels from 2010 to 2016 across the contiguous US. A notable concentration is seen in the southern regions. However, it also indicates that heat is a nationwide concern, with no census tracts completely void of caution+ heat days, emphasizing the universal exposure to substantial heat conditions across the country. No days of extreme danger level heat were recorded in the places where our sample lived, so this level is not shown.

**Fig. 3.**
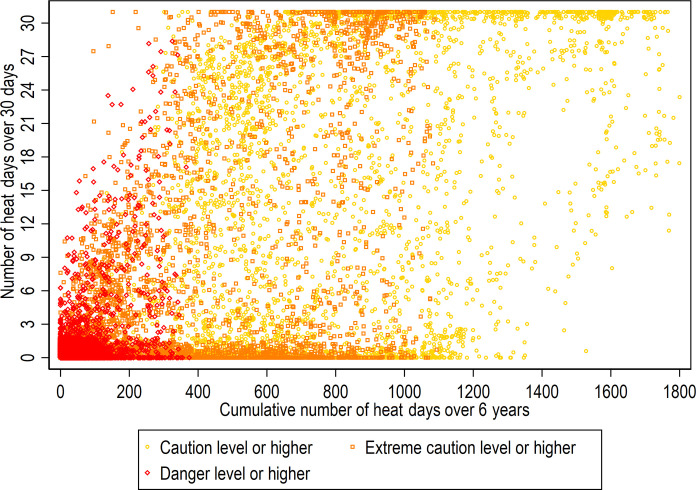
Distribution of heat days among study sample, 2016 health and retirement study venous blood study DNAm subsample.

Figure 3 provides a visualization of the distribution of heat days in the sample at different heat levels. The *x* axis represents the number of heat days over a 6-year period, and *y* axis shows the number of heat days over 30 days. At the caution+ level (yellow dots), the distribution of heat days is relatively wide. Extreme caution+ (orange dots) shows a similar but a sparser pattern in the middle range. At danger+ (red dots), the distribution is largely concentrated at lower numbers of heat days, although there is some coverage across all values (ranging from 0 to 430 heat days over 6 years) shown in the overall US distribution.

Table 2 summarizes the distribution of ambient heat days among the sample, categorized by caution+, extreme caution+, and danger+ levels over different time windows. Again, no days of extreme danger level heat are reported in our sample and thus not shown. On the BC day, 42.6% experience caution+ heat days in their neighborhoods, 18.6% facing extreme caution+, and 2% at danger+. Over a 1-year lagged time window, participants experience an average of 126.8 heat days of caution+ (SD = 68.3), 53.8 heat days at the extreme caution+ (SD = 51), and 6.1 heat days at the danger+ (SD = 12.7). Given the limited variation in the number of danger+ heat days, our subsequent analyses primarily consider the number of days at caution+ and extreme caution+ levels.

**Table 2.** Ambient heat across time windows among study sample. Heat measures include the total number of heat days between the BC date and the specified lagged time windows: the prior 7 days, 30 days, 60 days, 1 year, and a 6-year cumulative period. Estimates are weighted using the survey weights.

Lagged time window	Number of heat days		
	Caution+ level	Extreme caution+ level	Danger+ level
	Mean ± SD	Mean ± SD	Mean ± SD
BC day, %	42.6%	18.6%	2.0%
Lagged 7-day	3.3 days ± 3.4	1.5 days ± 2.6	0.2 days ± 0.8
Lagged 30-day	12.9 days ± 12.2	5.8 days ± 9.5	0.6 days ± 2.5
Lagged 60-day	25.7 days ± 22.8	11.7 days ± 18	1.4 days ± 5
Lagged 1-year	126.8 days ± 68.3	53.8 days ± 51	6.1 days ± 12.7
Cumulative (6-year)	741.4 days ± 380.7	314.6 days ± 283.7	39.5 days ± 62.3

Table S1 presents the differences in ambient outdoor heat days at caution+ and extreme caution+ levels across time windows by sociodemographic and health behavioral characteristics. Older adults, women, and nonheavy drinkers are found to reside in areas with more heat days over short- and mid-term periods. Over longer periods, individuals with lower wealth and those who do not engage in sufficient physical activity live in neighborhoods with more heat days. Non-Hispanic Blacks, Hispanics, individuals with lower education, and residents in socially vulnerable or suburban areas have more heat days across all time windows. This highlights significant sociodemographic group differences in outdoor heat profiles, with many of highly exposed groups also known to be at risk for accelerated biological aging, reinforcing the need to incorporate them as confounders in the models.

### Heat and epigenetic aging

**Fig. 4.**
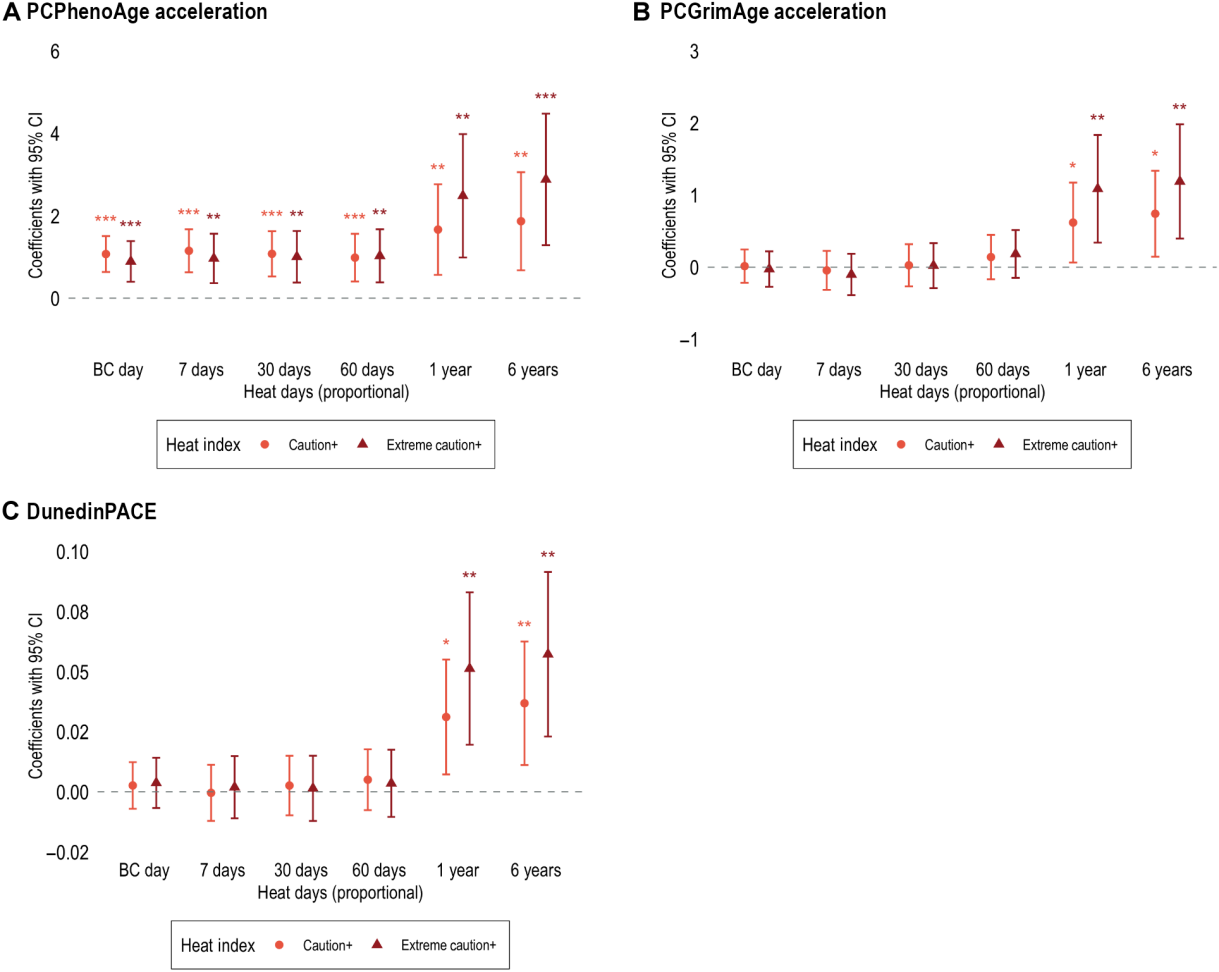
Association between ambient outdoor heat and accelerated epigenetic aging. *P < 0.05, **P < 0.01, and ***P < 0.001. The figures plot the estimated coefficients of ambient outdoor heat with 95% CIs at caution+ and extreme caution+ levels. Coefficients are derived from separate models where each epigenetic clock is regressed on each heat measure across two heat levels and six windows. *P* values are two-tailed and test the null hypothesis that the estimated *B* is equal to 0, based on *t* statistics. All models are adjusted for confounders that are potentially associated both outdoor heat and epigenetic clocks: cell types (i.e., %monocyte, %NK, %B, %CD8, and %CD4), age, sex, race/ethnicity, education, household wealth, smoking status, drinking status, obesity, physical activity, tract-level social vulnerability, urbanicity, and mean levels of O_3_ and PM_2.5_ for the same time windows used for outdoor heat measures. Table S2 shows the full suite of estimates. (**A**) From the models predicting PCPhenoAge Acceleration (**B**) PCGrimAge Acceleration (**C**) DunedinPACE.

Figure 4 displays the estimated effects of outdoor heat on epigenetic aging. Table S2 provides the full set of estimates. Results show significant associations between heat and accelerated epigenetic aging that differ across epigenetic clocks.

The number of heat days is associated with accelerated PCPhenoAge across all time windows and both levels of heat intensity. Individuals with area-level caution+ heat on their BC day show a 1.07-year increase in PCPhenoAge acceleration [95% confidence interval (CI) = 0.63, 1.51]. Since heat days are measured proportionally (ranging from 0 to 1) for lagged periods (i.e., 7, 30, 60 days, 1 year, and 6 years), a one-unit increase represents a shift from 0 to 100% heat days within the given period. Thus, a 10% increase in heat days corresponds to ^1^/_10_ of the reported coefficient. For example, one-unit increase in heat days at the caution+ level is associated with a 1.15-year increase in PCPhenoAge acceleration over a 7-day window (95% CI = 0.63, 1.67); this means that 10% more heat days corresponds to a 0.115-year PCPhenoAge acceleration. This association remains significant over 30-day (*B* = 1.08, 95% CI = 0.53, 1.62), 60-day (*B* = 0.98, 95% CI = 0.40, 1.56), 1-year (*B* = 1.66, 95% CI = 0.56, 2.77), and 6-year (*B* = 1.87, 95% CI = 0.68, 3.06) windows. Comparable results are observed for heat days at the extreme caution+ level; for example, a one-unit increase in heat days over a 6-year period is associated with a 2.88-year increase in PCPhenoAge acceleration (95% CI = 1.28, 4.48).

For PCGrimAge and DunedinPACE, significant heat effects are only found for longer time windows. In a 1-year window, a one-unit increase in heat days at the caution+ level is associated with a 0.62-year increase in PCGrimAge acceleration and a 3% faster DunedinPACE rate. Similarly, a one-unit increase in extreme caution+ heat days over a 6-year period corresponds to a 1.09-year increase in PCGrimAge acceleration and 5% faster DunedinPACE. Notably, associations are nonsignificant and near zero for short (BC day and 7-day) and mid-term (30-day and 60-day) windows.

### Heat and epigenetic aging and subgroup analyses

Figure S1 presents stratified models assessing the association between ambient heat and epigenetic aging across key sociodemographic subgroups, including age, gender, race/ethnicity, education, and wealth. The overall patterns remain largely consistent across subgroups. These findings do not provide strong evidence for heightened vulnerability or effect modification within specific sociodemographic groups.

### Sensitivity analysis

In general, the observed associations between heat and epigenetic aging are robust to a series of sensitivity analyses. We find consistent associations when using mean heat index (HI), calculated from daily mean air temperature and relative humidity (see table S3). Second, when we use natural cubic spline models, where we calculate the effect estimates at the 90th percentile of the number of heat days at the caution+ level compared to the 25th percentile, the findings remain the same (see table S4). Third, when we use average continuous HI values as a linear term in our models, the results are largely similar. One standardized unit increase in the HI values is significantly associated with epigenetic aging measures, following the same patterns shown in the main findings. We also identify an additional significant association between heat on the BC day with DunedinPACE (see table S5). Tables S2 to S5 also show the findings for the PCHorvathAge and PCHannumAge, where more heat days are associated primarily for the short-term time windows.

## DISCUSSION

This study makes several contributions to the growing body of research on the health implications of ambient outdoor heat. We examine whether short- (BC day and lagged 7 days), mid- (lagged 30 and 60 days), and longer-term (lagged 1 year and 6 years) heat is associated with epigenetic aging by leveraging a nationally representative sample of older adults merged with daily climate data covering the entire contiguous US based on respondents’ blood collection dates and places of residence. Our findings reveal significant associations between more heat days and accelerated epigenetic aging, particularly for longer-term periods. Specifically, we observe that short- and mid-term heat conditions are significantly associated with increases in PCPhenoAge, while more heat days more than 1 year and 6 years are linked to accelerated epigenetic aging in all epigenetic clocks.

The temporal patterns may reflect different magnitudes and types of biological responses to heat stress occurring in varying time frames. The observed short- and mid-term associations of heat on PCPhenoAge acceleration may be indicative of immediate physiological responses to heat stress. Previous research has identified specific methylation pathways that may potentially underlie these observations. In a preclinical mouse model, exposure to severe exertional heat stroke induced alterations in DNAm 30 days postexposure, predominantly characterized by hypermethylation (*8*). These changes were identified within genes associated with immunosuppression (*14*), in the promoter regions of cardiomyocytes genes implicated in stress responses (*15*), and in the transcriptional regulatory regions of genes relevant to skeletal muscle (*21*). In a human cohort, Bind *et al.* (*22*) analyzed nine candidate genes in blood samples from 777 older men in the Normative Aging Study and found that increased ambient temperature over the 3-week period was associated with toll-like receptor (TLR)-2 hypomethylation, a gene encoding a protein involved in innate immunity activation. The authors explained that this temperature-induced change in the TLR-2 gene methylation pattern may elevate interleukin-6 and C-reactive protein levels, indicating a potential inflammatory response to hot temperature exposures. Collectively, our results, within the context of existing literature, suggest that acute heat exposure may induce alterations in DNAm as maladaptive epigenetic responses, affecting downstream biological processes and accelerating aging.

We found consistent associations between long-term heat days and accelerated epigenetic aging across PCPhenoAge, PCGrimAge, and DunedinPACE. The short-term physiological effects of heat stress might not be stable and may last only a few days. Nonetheless, some effects could accumulate over extended periods, and it may be this continuous exposure that leads to methylation captured universally by the epigenetic clocks (*23*, *24*). This observation mirrors findings from prior studies showing robust associations and large effect sizes for longer-term heat exposure on accelerated epigenetic aging (*19*, *20*) and cytosine-phosphate-guanine (CpG)-specific DNAm (*23*). For instance, a study in Germany found associations between annual average temperatures and multiple epigenetic clocks examined, while there were fewer associations with a shorter 8-week window (*19*). Similarly, a study in Taiwan identified that effect sizes for epigenetic aging increased with heat exposure durations from 1 to 180 days (*20*). Furthermore, genome-wide DNAm analyses documented that, among the 31 CpGs significantly associated with ambient temperature, the strength of temperature-methylation associations increased with longer exposure periods (*23*). Moreover, residing in an area with extended heat conditions can shape one’s psychosocial outcomes and health behaviors closely related to aging, which less likely result from temporary heat exposure. For example, persistent exposure to high temperatures can elevate stress and anxiety due to frequent sleep disruptions and physical discomfort (*25*) and may reduce overall physical activity levels (*26*), all of which can contribute to faster health decline with age. This suggests that prolonged ambient heat can drive more persistent and extensive changes in physiological deterioration that accumulate over time and are reflected in accelerated biological aging.

Another source of variation across epigenetic clocks stems from the different selection of CpG sites, with minimum overlap among them (*27*). This distinct selection of CpG sites across clocks highlights the unique biological aging aspects each clock may capture and their differential sensitivities to environmental stressors (*28*). Prior study on temperature and genome-wide DNAm among 479 Australian women reported that ambient temperatures during varying time windows (from 1 day to 1 year) were associated with DNAm levels of different genes, with only few overlaps between short and long exposure windows (*23*). Of the 22 genes whose methylation levels were linked with 1-year temperature exposure, only two genes’ methylation was associated with temperature over shorter exposure windows. Therefore, it is possible that the CpG sites integrated into the PCPhenoAge may capture a broader spectrum of the biological aging processes affected by heat stress, including changes in the body’s internal environment induced by both acute and chronic heat stress. On the other hand, CpG sites selected in PCGrimAge and DunedinPACE could be more sensitive to the system-wide physiological dysregulation accumulating over extended periods to heat exposure. To summarize, our findings of different relationships between heat and epigenetic aging across clocks may suggest a role of different CpG sites varying in response to heat. Our understanding of the differential patterns across clocks would be improved with further investigation of the specific CpG sites that account for the differential responses of the clocks to different types and windows of heat exposure.

This study examines the association between heat and epigenetic aging in a nationally representative, diverse sample of older adults. This marks a significant advancement beyond prior research, which predominantly relied on regionally selective samples lacking racial/ethnic and socioeconomic representation, thus enhancing the potential for broader population-level applicability of the findings. Second, this study uses high spatiotemporal resolution daily climatic parameters covering the entire contiguous US at the census-tract level and considers respondents’ relocations. This approach allowed for more accurate creation of both short-term heat measure near BC dates and cumulative heat measure since the baseline of 2010, minimizing misclassification due to relocation. Third, our work distinguishes between epigenetic clocks responsive to immediate heat events and those affected by prolonged heat. By incorporating data on 6-year cumulative ambient heat conditions, our study further uncovers potential dose-response effects that shorter-term analyses might overlook, offering insights into the cumulative health implications of heat.

The interpretation of our results must be considered within the context of several limitations (see appendix S1 for an extended version). First, our data did not have repeated measurements of epigenetic clocks, which did not allow us to observe and analyze longitudinal changes in response to ambient outdoor heat. Second, despite incorporating a comprehensive set of covariates to account for potential confounding factors, the observational nature of this study cannot entirely rule out the influence of residual confounding or the impact of unmeasured variables such as amount of time respondents spend outdoors and use of temperature control within their home. Therefore, it is crucial to interpret our results as reflecting the potential for heat exposure rather than direct, personal heat exposure. Third, our analysis used census tract-level HI values rather than a more spatially refined geographic scope (e.g., block level). Census tracts can encompass diverse areas different in land use, vegetation, and built environment, all of which can affect local temperatures. Thus, there is a potential for underestimating the true local heat conditions for some individuals, particularly those living in areas that are hotter than the tract average due to microclimate effects (*29*). Fourth, the Health and Retirement Study (HRS) does not regularly collect information on respondents’ access to or use of air conditioning, so we were not able to directly account for its potential role in mitigating outdoor heat effects. Fifth, the validity of epigenetic clocks across genetically and environmentally diverse populations has yet to be established (*30*). As population-specific clocks are not yet widely available, we were unable to adjust our analyses for these genetic and environmental variations. Sixth, it is important to acknowledge that epigenetic modifications may represent adaptive responses to heat through acclimation or acclimatization rather than solely maladaptive changes associated with accelerated aging. The current study’s observational design does not allow us to definitively distinguish between these adaptive and maladaptive processes.

Our study provides insights into the biological underpinnings linking heat to the broader spectrum of aging-related morbidity and mortality risks. We demonstrated that short-, mid-, and long-term ambient outdoor heat can significantly accelerate epigenetic aging within a diverse, nationally representative cohort of older adults. This provides strong evidence critical for guiding public policy and advocacy initiatives aimed at developing mitigation strategies against climate change. Furthermore, our findings serve as a foundation for the development of targeted public health interventions, providing a strategic framework for addressing the adverse biological impacts triggered by extreme heat.

## MATERIALS AND METHODS

### Data

This study use data from the HRS 2016 Venous Blood Study (VBS) DNAm subsample (*N* = 3875). HRS is a US nationally representative longitudinal study of older adults, replenishing the sample every 6 years with younger birth cohorts (*31*). All participants in the 2016 HRS wave, except for nursing home residents and proxy respondents, were invited to the VBS. Verbal informed consent was obtained from all VBS participants. Among them, DNAm assays were conducted on a nonrandom subsample. HRS provides weights for the DNAm subsample to adjust for the differential selection and rates of participation (*32*). When weighted, this subsample represents the entire community-dwelling HRS sample aged 56 and older, encompassing the racial/ethnic and socioeconomic diversity of the US older population (*18*). As shown in fig. S2, this subsample is distributed across all nine US Census Division, demonstrating its geographic representation. To assess heat, we use meteorological data from gridMet (*33*), which has daily weather information (e.g., maximum and minimum values of temperature and relative humidity) at a spatial resolution of 4 km by 4 km for the contiguous US. We calculate a daily HI value for each grid based on a formula from the National Weather Service (NWS) HI (*34*). We estimate values at the geographical boundaries of census tracts by taking an area-weighted average of the gridded data. In cases where a single grid covers an entire census tract, we directly use the corresponding HI values from that grid. When a grid crossed a tract boundary, meaning multiple grids overlapped a single census tract, we calculate the HI for the tract by averaging the values from the intersecting grids, weighted by the area of each grid section within the tract. Then, we link heat data to respondents in the HRS DNAm sample using the census tract to which their address was geocoded and their BC date. In addition, we obtain 2018 Centers for Disease Control and Prevention (CDC) Social Vulnerability Index (SVI) at the census tract level (*35*). We further obtain census tract daily PM_2.5_ and O_3_ concentrations from the Environmental Protection Agency’s Fused Air Quality Surface Downscaling Model that integrates daily air quality monitoring data with modeled outputs (*36*), which are available via the HRS-Contextual Data Resource (CDR) (*37*).

The final analytic sample consists of 3686 respondents, after excluding participants with missing data on geographic identifies (*n* = 30), heat measures (*n* = 1), and covariates (*n* = 158). For analyses of 6-year cumulative heat, the sample is slightly reduced to 3679 after excluding 7 respondents with incomplete geographic information over time. A detailed sample selection process is described in fig. S3. We compare the final analytic sample to the excluded respondents with incomplete data (*n* = 189) by creating a group indicator for missing data status. We test differences between the two groups using linear regressions for continuous variables and Pearson χ^2^ tests for categorical variables. As shown in table S6, the group with missing data has a higher proportion of non-Hispanic Black and Hispanic respondents and fewer years of education. No significant differences are observed in other sociodemographic factors, health behavioral characteristics, or epigenetic clocks.

### Measures

#### Ambient outdoor heat

We use the HI as a measure of heat for several reasons. First, the HI approximates apparent temperature by incorporating both air temperature and relative humidity to reflect what the temperature feels like to the human body. Physiological studies have shown that increasing humidity along with increasing temperature exacerbates the heat-induced thermoregulatory strain because high humidity impairs the rate of evaporation from the body, a key mechanism for cooling down (*38*, *39*). Aging is associated with impaired thermoregulatory control due to diminished function of sweat glands and reduced skin blood flow responses as skin ages (*40*). This underscores the importance of accounting for humidity in a study of heat effects among older adults. Second, the HI is designed to estimate heat stress on the human body in shaded environments, which is more suitable for the day-to-day activities (*41*) older adults are likely to engage in.

We calculate gridded ambient heat based on the NWS HI equation. For detailed information on the base equation and adjustment methods, please see appendix S2 (*42*). Solar radiation, windiness, clothing resistance, and human physiology are implied in the equation through multiple regression analyses based on results produced by the Steadman’s heat budget model (*43*). Following the work by Dahl *et al.* (*34*), we calculate the daily maximum HI from the daily maximum ambient temperature (°F) and the minimum relative humidity (%). The HI is presented as an apparent temperature in degrees Fahrenheit (°F). Increasing (or decreasing) humidity results in different HI values with the same ambient temperature. For example, at an ambient temperature of 100°F (37.3°C), a relative humidity of 55% corresponds to a HI of 124°F (51.1°C), while a relative humidity of 15% results in a HI of 96°F (35.5°C). This indicates that at lower relative humidity, the apparent temperature can be lower than the air temperature.

Then, HI values are classified into categories to evaluate the potential risk of adverse health effects, in accordance with the NWS guidelines (*44*). HI values ranging from 80° to 90°F (26.7° to 32.2°C) is classified as the caution level, signaling an escalating concern for heat-related health conditions. HI values between 90° and 103°F (39.4° and 32.2°C) is considered to be at the extreme caution level, indicative of a moderate risk of heat-related health impacts, particularly among vulnerable populations. The danger level encompasses HI values between 103° and 124°F (32.3° and 51.1°C) and signifies a high risk of heat-related health impacts across the general population, especially if adequate cooling measures are not undertaken. Any HI value above 124°F (51.1°C) falls into the extreme danger level, which denotes a critical risk for heat-related health impacts. However, none of our sample lived in places where extreme danger level heat was recorded, and therefore, no estimates for this category are included in the results.

To assess the effects of varying durations of outdoor heat conditions, we create count measures (the total number of heat days) reflecting several lagged time windows: the BC day, lagged 7 days, 30 days, 60 days, 1 year, and 6 years before their blood collection. Short-term time windows are defined as the BC day and the preceding 7-day period. This allows for the investigation of how epigenetic aging is related to acute heat stressors. Mid-term time windows, encompassing several weeks, align with the temporal scope needed to observe physiological and biological delayed responses to temperature conditions. Longer-term heat assessments (1 year and 6 years) capture the chronic climate conditions individuals experience. Such longitudinal evaluation facilitates an understanding of the enduring impacts of heat over extended periods, potentially exerting more pronounced effects on health trajectories and aging processes. Our analysis is limited to a maximum 6-year cumulative window for longer heat conditions because the baseline interview for about 30% occurred in 2010 and prior residential address information is not available. These time frames are evaluated for each of the HI categories. For the BC day heat, we create a binary variable indicating if the day’s HI fell into caution, extreme caution, and danger levels. For the lagged time window, we calculate the total number of heat days that reached each heat level within each time window. For instance, if one’s blood was collected on 8 July 2016, then their 7-day lagged heat would encompass the total number of heat days from 1 July to 8 July, inclusive of the BC day.

About 22% of our sample relocated to different census tracts during the study period. To account for this residential mobility, we combine cross-wave census tract information from the sample, verified from the biennial HRS surveys, with self-reported data on when the respondent moved to a new residence. Specifically, when current census tract information differs from prior waves, we estimate the timing of the move using the year and month of the most recent move reported by respondents. In the case where moving date is not reported, we estimate the move date as the midpoint between interviews. We then link HI data to the corresponding census tract. For example, if a participant lived in census tract A until March 2014 and moved to tract B in April 2014, we use HI data from tract A until March 2014 and from tract B thereafter. To ensure comparability across the different time windows, we calculate the proportion of days classified as “heat days” within each timeframe, standardizing measures on a scale from 0 to 1. As an example, for the 1-year window that includes the BC day and the preceding 365 days, we divided the total number of heat days by 366, allowing for a possible maximum value of 1. Higher values on this metric indicate greater heat days over the specified period.

#### Epigenetic clocks

DNAm was assessed from peripheral blood cell DNA extracted from the buffy coat using the Infinium Methylation EPIC BeadChip microarrays. DNAm epigenetic age measures, or epigenetic clocks, have been estimated from the genome-wide methylation β values. For detailed methodologies, including data preprocessing and quality control, please see the study of Crimmins *et al.* (*45*). Our study focuses on three DNAm epigenetic age measures now used in population health research: PhenoAge (*27*), GrimAge (*46*), and DunedinPACE (*47*). PhenoAge and GrimAge are second-generation measures trained to better predict health outcomes by incorporating age-associated biomarkers (e.g., C-reactive protein), behaviors (e.g., smoking), and health outcomes (e.g., mortality). For PhenoAge and GrimAge, epigenetic age is represented in years. We calculate age acceleration by taking the residual from regressing epigenetic age on chronological age. The residual represents the deviation of epigenetic age from chronological age with the values greater than zero indicating faster epigenetic aging in unit of year. For GrimAge acceleration, the residual is obtained from regressing GrimAge on chronological age and sex because both age and sex are part of its original calculation. We also use DunedinPACE, a third-generation measure, which is designed to predict within-individual changes in biomarkers and health outcomes as the predicted phenotype. It quantifies the pace of aging in terms of years of physiological decline occurring per calendar year. PhenoAge and GrimAge are further trained through principal components (PC) analysis to minimize the effect of noise from any single CpG (*48*), hereafter termed PCPhenoAge and PCGrimAge. The DunedinPACE measure is already optimized by removing unreliable CpG sites, and thus additional PC training is not required. We also examine PCHorvathAge (*49*) and PCHannumAge (*50*), the first-generation epigenetic clocks trained to predict chronological age. However, given prior research documenting their weaker prediction of health and mortality outcomes and less reliable associations with social exposures compared to the second- and third- generation clocks (*30*), we include these findings only in the Supplementary Materials. Figure S4 shows the distributions of epigenetic aging measures.

Control variables are selected a priori, based on their known association with ambient heat and epigenetic aging. Individual-level covariates include *cell type composition* [%monocyte, %natural killer (NK), %B, %CD8, and %CD4], *age in years*, *gender* (men and women), *race/ethnicity* (non-Hispanic white, non-Hispanic Black, Hispanic, and non-Hispanic Other), *education in years* (top-coded at 17 or more due to high kurtosis), *household wealth* (total nonhousing wealth transformed using an inverse hyperbolic sine), and health behaviors [*smoking* (never, former, and current), *alcohol use* (noncurrent, light, and heavy drinking—7 days a week), *obesity* (body mass index ≥ 30), and *physical activity* (sufficient versus nonsufficient)]. For physical activity, we use self-report data on how often they engage in moderate-intensity (e.g., dancing and walking) and vigorous-intensity (e.g., running and swimming) physical activities in their daily lives. Response options included “never,” “1 to 3 times per month,” “once per week,” “more than once per week,” and “every day.” On the basis of the World Health Organization’s guidelines for older adults (at least 150 min of moderate physical activity or 75 min of vigorous activity per week) (*51*), those engaging in moderate activity more than once a week or vigorous activity at least once a week are classified as having sufficient physical activity levels. In addition, the model accounts for area-level characteristics to control for potential confounding effects arising from variations in the frequency of heat days and health conditions. These include *urbanicity* (urban, sub-urban, and ex-urban) and the *CDC SVI* at the census tract level that ranks all US census tracts based on 15 social factors such as socioeconomic status, housing, and minority composition. Each tract receives a percentile rank ranging from 0 to 1, with higher values representing greater relative vulnerability. We use the overall tract summary ranking. For further details, please refer to the CDC SVI documentation (*35*). Last, we adjust for the mean levels of *O*_*3*_ and *PM*_*2.5*_ at the census tract over the same time windows used for heat to adjust for the potential confounding effects from air pollution.

### Statistical analysis

We use multilevel linear regression models that account for the hierarchical nature of data, with individuals (level one) nested within census tracts and a proxy for neighborhoods (level two). This approach adjusts for the nonindependence of observations from persons clustered within neighborhoods when assessing the association between extreme heat and accelerated epigenetic aging. We fit a series of separate models to test the association of heat with epigenetic clocks for each combination of heat levels, time frame, and outcome. Specifically, we analyze two heat levels (caution+ and extreme caution+) across six different time windows for five epigenetic clocks, resulting in a total of 60 models. All models are adjusted for previously mentioned covariates. Appendix S3 presents the full model specifications.

There are 320 individuals (8.7% of the final analytic sample) missing data for one or more cell type compositions: NK cells (*n* = 233), monocytes (*n* = 233), B cells (*n* = 212), CD8 T cells (*n* = 212), and CD4 T cells (*n* = 212). Details of missing data patterns are provided in fig. S5. These missing values are imputed with multiple imputations by chained equations. Sociodemographic variables (i.e., age, gender, race/ethnicity, education, and household wealth) are included in linear regression models to predict cell type compositions. We generate 10 imputed datasets, and the parameters and SEs across from these datasets are combined using Rubin’s rules. All analyses apply VBS DNAm-specific weights to account for the differential probabilities of the selection into and participation in the HRS VBS, making the estimates population representative. Specifically, these respondent-level weights are included at level 1 of all multilevel models. We also use the pwscale(size) option in Stata to account for the hierarchical data structure. All analyses are conducted in Stata 18.0, with some figures produced using R.

We conduct several sensitivity analyses to evaluate the robustness of our findings. First, to address variability in temperature and humidity throughout the day, we calculate an alternative HI using the average of the daily maximum and minimum values for both temperature and relative humidity and then repeat the analyses with this mean HI. Second, to account for potential nonlinear associations between the number of heat days and epigenetic aging, we use spline models. We model these associations using natural cubic splines with three knots. The Bayesian information criterion and Akaike information criterion demonstrate incremental increases with additional knots, suggesting that a three-knot model offers an optimal balance of complexity and parsimony. The effect estimates represent in epigenetic clocks per increase in heat days at the caution+ level from the 25th to the 90th percentiles. Third, we use standardized average continuous HI values over specified time windows as a linear term in our models, instead of the number of heat days.

## References

[1] W. Marx, R. Haunschild, L. Bornmann, Heat waves: A hot topic in climate change research. Theor. Appl. Climatol. 146, 781–800 (2021). 34493886 10.1007/s00704-021-03758-y PMC8414451

[2] F. Batibeniz, M. Ashfaq, N. S. Diffenbaugh, K. Key, K. J. Evans, U. U. Turuncoglu, B. Önol, Doubling of U.S. population exposure to climate extremes by 2050. Earths Future 8, e2019EF001421 (2020).

[3] J. B. Layton, W. Li, J. Yuan, J. P. Gilman, D. B. Horton, S. Setoguchi, Heatwaves, medications, and heat-related hospitalization in older Medicare beneficiaries with chronic conditions. PLOS ONE 15, e0243665 (2020). 33301532 10.1371/journal.pone.0243665 PMC7728169

[4] S. E. Cleland, W. Steinhardt, L. M. Neas, J. Jason West, A. G. Rappold, Urban heat island impacts on heat-related cardiovascular morbidity: A time series analysis of older adults in US metropolitan areas. Environ. Int. 178, 108005 (2023). 37437316 10.1016/j.envint.2023.108005 PMC10599453

[5] S. A. M. Khatana, R. M. Werner, P. W. Groeneveld, Association of extreme heat with all-cause mortality in the contiguous US, 2008-2017. JAMA Netw. Open 5, e2212957 (2022). 35587347 10.1001/jamanetworkopen.2022.12957 PMC9121188

[6] E. J. W. Van Someren, Thermoregulation and aging. Am. J. Physiol. Regul. Integr. Comp. Physiol. 292, R99–R102 (2007). 16902181 10.1152/ajpregu.00557.2006

[7] E. Crimmins, J. K. Kim, S. Vasunilashorn, Biodemography: New approaches to understanding trends and differences in population health and mortality. Demography 47, S41–S64 (2010). 21302421 10.1353/dem.2010.0005 PMC5870619

[8] K. O. Murray, T. L. Clanton, M. Horowitz, Epigenetic responses to heat: From adaptation to maladaptation. Exp. Physiol. 107, 1144–1158 (2022). 35413138 10.1113/EP090143 PMC9529784

[9] D. C. H. Metzger, P. M. Schulte, Persistent and plastic effects of temperature on DNA methylation across the genome of threespine stickleback (*Gasterosteus aculeatus*). Proc. R. Soc. Lond. B Biol. Sci. 284, 20171667 (2017). 10.1098/rspb.2017.1667 PMC5647309 28978736

[10] C. L. Martin, L. Ghastine, E. K. Lodge, R. Dhingra, C. K. Ward-Caviness, Understanding health inequalities through the lens of social epigenetics. Annu. Rev. Public Health 43, 235–254 (2022). 35380065 10.1146/annurev-publhealth-052020-105613 PMC9584166

[11] Q.-L. Wan, X. Meng, W. Dai, Z. Luo, C. Wang, X. Fu, J. Yang, Q. Ye, Q. Zhou, N6-methyldeoxyadenine and histone methylation mediate transgenerational survival advantages induced by hormetic heat stress. Sci. Adv. 7, eabc3026 (2021). 33523838 10.1126/sciadv.abc3026 PMC7775758

[12] A. Vinoth, T. Thirunalasundari, M. Shanmugam, A. Uthrakumar, S. Suji, U. Rajkumar, Evaluation of DNA methylation and mRNA expression of heat shock proteins in thermal manipulated chicken. Cell Stress Chaperones 23, 235–252 (2018). 28842808 10.1007/s12192-017-0837-2 PMC5823805

[13] A. Weyrich, S. Benz, S. Karl, M. Jeschek, K. Jewgenow, J. Fickel, Paternal heat exposure causes DNA methylation and gene expression changes of *Stat3* in Wild guinea pig sons. Ecol. Evol. 6, 2657–2666 (2016). 27066228 10.1002/ece3.1993 PMC4769883

[14] K. O. Murray, J. O. Brant, J. D. Iwaniec, L. H. Sheikh, L. de Carvalho, C. K. Garcia, G. P. Robinson, J. M. Alzahrani, A. Riva, O. Laitano, M. P. Kladde, T. L. Clanton, Exertional heat stroke leads to concurrent long-term epigenetic memory, immunosuppression and altered heat shock response in female mice. J. Physiol. 599, 119–141 (2021). 33037634 10.1113/JP280518

[15] K. O. Murray, J. O. Brant, M. P. Kladde, T. L. Clanton, Long-term epigenetic and metabolomic changes in the mouse ventricular myocardium after exertional heat stroke. Physiol. Genomics 54, 486–500 (2022). 36215393 10.1152/physiolgenomics.00147.2021 PMC9705024

[16] A. Cardenas, R. Fadadu, S. Bunyavanich, Climate change and epigenetic biomarkers in allergic and airway diseases. J. Allergy Clin. Immunol. 152, 1060–1072 (2023). 37741554 10.1016/j.jaci.2023.09.011 PMC10843253

[17] R. Xu, S. Li, S. Guo, Q. Zhao, M. J. Abramson, S. Li, Y. Guo, Environmental temperature and human epigenetic modifications: A systematic review. Environ. Pollut. 259, 113840 (2020). 31884209 10.1016/j.envpol.2019.113840

[18] E. Crimmins, B. Thyagarajan, M. E. Levine, D. R. Weir, J. Faul, Associations of age, sex, race/ethnicity, and education with 13 epigenetic clocks in a nationally representative U.S. sample: The health and retirement study. J. Gerontol. A Biol. Sci. Med. Sci. 76, 1117–1123 (2021). 33453106 10.1093/gerona/glab016 PMC8140049

[19] W. Ni, N. Nikolaou, C. K. Ward-Caviness, S. Breitner, K. Wolf, S. Zhang, R. Wilson, M. Waldenberger, A. Peters, A. Schneider, Associations between medium- and long-term exposure to air temperature and epigenetic age acceleration. Environ. Int. 178, 108109 (2023). 37517177 10.1016/j.envint.2023.108109 PMC10656697

[20] K.-C. Chiu, M.-S. Hsieh, Y.-T. Huang, C.-Y. Liu, Exposure to ambient temperature and heat index in relation to DNA methylation age: A population-based study in Taiwan. Environ. Int. 186, 108581 (2024). 38507934 10.1016/j.envint.2024.108581

[21] K. Murray, L. Sheikh, O. Laitano, J. Iwaniec, C. Garcia, G. Robinson, R. Hammamieh, R. Campbell, R. Yang, T. Clanton, Epigenetic memory and phenotype change observed in mouse skeletal muscle 30 days after exertional heat stroke. FASEB J. 33, 842.5-842.5 (2019).

[22] M.-A. Bind, A. Zanobetti, A. Gasparrini, A. Peters, B. Coull, A. Baccarelli, L. Tarantini, P. Koutrakis, P. Vokonas, J. Schwartz, Effects of temperature and relative humidity on DNA methylation. Epidemiology 25, 561–569 (2014). 24809956 10.1097/EDE.0000000000000120 PMC4224120

[23] R. Xu, S. Li, S. Li, E. M. Wong, M. C. Southey, J. L. Hopper, M. J. Abramson, Y. Guo, Ambient temperature and genome-wide DNA methylation: A twin and family study in Australia. Environ. Pollut. 285, 117700 (2021). 34380236 10.1016/j.envpol.2021.117700

[24] R. Chen, P. Yin, L. Wang, C. Liu, Y. Niu, W. Wang, Y. Jiang, Y. Liu, J. Liu, J. Qi, J. You, H. Kan, M. Zhou, Association between ambient temperature and mortality risk and burden: Time series study in 272 main Chinese cities. BMJ 363, k4306 (2018). 30381293 10.1136/bmj.k4306 PMC6207921

[25] M. K. K. Rony, H. M. Alamgir, High temperatures on mental health: Recognizing the association and the need for proactive strategies—A perspective. Health Sci. Rep. 6, e1729 (2023). 38059052 10.1002/hsr2.1729 PMC10696165

[26] J. Y. Ho, H. Y. C. Lam, Z. Huang, S. Liu, W. B. Goggins, P. K. H. Mo, E. Y. Y. Chan, Factors affecting outdoor physical activity in extreme temperatures in a sub-tropical Chinese urban population: An exploratory telephone survey. BMC Public Health 23, 101 (2023). 36641429 10.1186/s12889-022-14788-0 PMC9840260

[27] M. E. Levine, A. T. Lu, A. Quach, B. H. Chen, T. L. Assimes, S. Bandinelli, L. Hou, A. A. Baccarelli, J. D. Stewart, Y. Li, E. A. Whitsel, J. G. Wilson, A. P. Reiner, A. Aviv, K. Lohman, Y. Liu, L. Ferrucci, S. Horvath, An epigenetic biomarker of aging for lifespan and healthspan. Aging 10, 573–591 (2018). 29676998 10.18632/aging.101414 PMC5940111

[28] D. J. Simpson, T. Chandra, Epigenetic age prediction. Aging Cell 20, e13452 (2021). 34415665 10.1111/acel.13452 PMC8441394

[29] D. T. O’Brien, B. Gridley, A. Trlica, J. A. Wang, A. Shrivastava, Urban heat islets: Street segments, land surface temperatures, and medical emergencies during heat advisories. Am. J. Public Health 110, 994–1001 (2020). 10.2105/AJPH.2020.305636 PMC7287541 32437273

[30] E. M. Crimmins, E. T. Klopack, J. K. Kim, Generations of epigenetic clocks and their links to socioeconomic status in the Health and Retirement Study. Epigenomics 16, 1031–1042 (2024). 39023350 10.1080/17501911.2024.2373682 PMC11404624

[31] A. Sonnega, J. D. Faul, M. B. Ofstedal, K. M. Langa, J. W. Phillips, D. R. Weir, Cohort profile: The Health and Retirement Study (HRS). Int J Epidemiol 43, 576–585 (2014). 24671021 10.1093/ije/dyu067 PMC3997380

[32] J. D. Faul, E. Crimmins, S. Munro, B. Thyagarajan, D. Weir, HRS DNA Methylation Data–VBS 2016. (2023). https://hrs.isr.umich.edu/sites/default/files/genetic/HRS_DNAm_OCT2023.pdf.

[33] John T. Abatzoglou Climatology Lab, University of California Merced, gridMET, *Climatology Lab* (2023). https://climatologylab.org/gridmet.html.

[34] K. Dahl, R. Licker, J. T. Abatzoglou, J. Declet-Barreto, Increased frequency of and population exposure to extreme heat index days in the United States during the 21st century. Environ. Res. Commun. 1, 075002 (2019).

[35] Centers for Disease Control and Prevention/ Agency for Toxic Substances and Disease Registry/ Geospatial Research, Analysis, and Services Program., CDC/ATSDR Social Vulnerability Index 2018 Database U.S.; https://atsdr.cdc.gov/placeandhealth/svi/data_documentation_download.html.

[36] US EPA, Technical Information about Fused Air Quality Surface Using Downscaling Tool: Metadata Description (2016). https://www.epa.gov/sites/production/files/2016-07/documents/data_fusion_meta_file_july_2016.pdf.

[37] J. Ailshire, S. Mawhorter, E. Y. Choi, “Contextual Data Resource (CDR): US Decennial Census and American Community Survey Data, 1990-2018, Version 2.0.” (USC/UCLA Center on Biodemography and Population Health, Los Angeles, 2020); https://hrs.isr.umich.edu/sites/default/files/restricted_data_docs/Census_ACS_HRS-CDR_Documentation_2020-09-14.pdf.

[38] R. G. Steadman, The assessment of sultriness. Part I: A temperature-humidity index based on human physiology and clothing science. J. Appl. Meteorol. 18, 861–873 (1979).

[39] S. C. Sherwood, M. Huber, An adaptability limit to climate change due to heat stress. Proc. Natl. Acad. Sci. U.S.A. 107, 9552–9555 (2010). 20439769 10.1073/pnas.0913352107 PMC2906879

[40] B. N. Balmain, S. Sabapathy, M. Louis, N. R. Morris, Aging and thermoregulatory control: The clinical implications of exercising under heat stress in older individuals. Biomed. Res. Int. 2018, 8306154 (2018). 30155483 10.1155/2018/8306154 PMC6098859

[41] National Weather Service, Wet Bulb Globe Temperature vs Heat Index.

[42] National Weather Service, Heat Index Equation (2022). https://wpc.ncep.noaa.gov/html/heatindex_equation.shtml.

[43] L. P. Rothfusz, The Heat Index “Equation” (or, More Than You Ever Wanted to Know About Heat Index) (1990).

[44] N. US Department of Commerce, What is the heat index? https://weather.gov/ama/heatindex.

[45] E. Crimmins, J. K. Kim, J. Fisher, J. Faul, HRS Epigenetic Clocks (2020). https://hrsdata.isr.umich.edu/sites/default/files/documentation/data-descriptions/EPICLOCKS_DD.pdf.

[46] A. T. Lu, A. Quach, J. G. Wilson, A. P. Reiner, A. Aviv, K. Raj, L. Hou, A. A. Baccarelli, Y. Li, J. D. Stewart, E. A. Whitsel, T. L. Assimes, L. Ferrucci, S. Horvath, DNA methylation GrimAge strongly predicts lifespan and healthspan. Aging 11, 303–327 (2019). 30669119 10.18632/aging.101684 PMC6366976

[47] D. W. Belsky, A. Caspi, D. L. Corcoran, K. Sugden, R. Poulton, L. Arseneault, A. Baccarelli, K. Chamarti, X. Gao, E. Hannon, H. L. Harrington, R. Houts, M. Kothari, D. Kwon, J. Mill, J. Schwartz, P. Vokonas, C. Wang, B. S. Williams, T. E. Moffitt, DunedinPACE, a DNA methylation biomarker of the pace of aging. eLife 11, e73420 (2022). 35029144 10.7554/eLife.73420 PMC8853656

[48] A. T. Higgins-Chen, K. L. Thrush, Y. Wang, C. J. Minteer, P.-L. Kuo, M. Wang, P. Niimi, G. Sturm, J. Lin, A. Z. Moore, S. Bandinelli, C. H. Vinkers, E. Vermetten, B. P. F. Rutten, E. Geuze, C. Okhuijsen-Pfeifer, M. Z. van der Horst, S. Schreiter, S. Gutwinski, J. J. Luykx, M. Picard, L. Ferrucci, E. M. Crimmins, M. P. Boks, S. Hägg, T. T. Hu-Seliger, M. E. Levine, A computational solution for bolstering reliability of epigenetic clocks: Implications for clinical trials and longitudinal tracking. Nat. Aging 2, 644–661 (2022). 36277076 10.1038/s43587-022-00248-2 PMC9586209

[49] S. Horvath, DNA methylation age of human tissues and cell types. Genome Biol. 14, R115 (2013). 24138928 10.1186/gb-2013-14-10-r115 PMC4015143

[50] G. Hannum, J. Guinney, L. Zhao, L. Zhang, G. Hughes, S. Sadda, B. Klotzle, M. Bibikova, J.-B. Fan, Y. Gao, R. Deconde, M. Chen, I. Rajapakse, S. Friend, T. Ideker, K. Zhang, Genome-wide methylation profiles reveal quantitative views of human aging rates. Mol. Cell 49, 359–367 (2013). 23177740 10.1016/j.molcel.2012.10.016 PMC3780611

[51] World Health Organization, “Global Recommendations on Physical Activity for Health” (WHO Press, World Health Organization, 2010); https://iris.who.int/handle/10665/44399. 26180873

