# Supplementary material for: Ambient outdoor heat and accelerated epigenetic aging among older adults in the US
Source: Sci Adv. 2025 Feb 26;11(9):eadr0616. doi: 10.1126/sciadv.adr0616 (PMC11864172; doi:10.1126/sciadv.adr0616)
Supplement: Supplementary file 1 — Appendix S1 to S3 Figs. S1 to S5 Tables S1 to S6 [file sciadv.adr0616_sm.pdf]

Science Advances
AAAS

Supplementary Materials for

Ambient outdoor heat and accelerated epigenetic aging among older adults in the US

Eun Young Choi, and Jennifer A. Ailshire

Corresponding author: Eun Young Choi, choieuny@usc.edu

Sci. Adv. **11**, eadr0616 (2025)
DOI: 10.1126/sciadv.adr0616

This PDF file includes:

Appendix S1 to S3
Figs. S1 to S5
Tables S1 to S6

Appendix S1. Extended Description on Study Limitations

The interpretation of our results must be considered within the context of several limitations. First, our data did not have repeated measurements of epigenetic clocks, which did not allow us to observe and analyze longitudinal changes in response to ambient outdoor heat. Future research with longitudinal data is essential to determine whether outdoor heat is associated with sustained, faster changes in epigenetic aging and to improve the causal inference in the association observed in our study. Second, despite incorporating a comprehensive set of covariates to account for potential confounding factors, the observational nature of this study cannot entirely rule out the influence of residual confounding or the impact of unmeasured variables. These factors include the amount of time respondents spend outdoors, access and use of temperature control within their home, and intake of certain medications such as vasodilators that can increase one's susceptibility to heat. Therefore, it is crucial to interpret our results as reflecting the *potential* for heat exposure, rather than direct, personal heat exposure. Third, our analysis used census tract-level outdoor heat index values to estimate exposure, rather than a more spatially-refined geographic scope (e.g., block-level). Census tracts can encompass diverse areas different in land use, vegetation, and built environment, all of which can affect local temperatures. Thus, there is a potential for underestimating the true heat exposure for some individuals, particularly those living in areas that are hotter than the tract average due to microclimate effects. For example, prior research has introduced the concept of *urban heat islets* – individual streets substantially warmer than their surrounding areas – by demonstrating substantial variance in land surface temperature between streets within the same census tracts. Fourth, the HRS does not regularly collect information on respondents' access to or use of air conditioning. Although we included a wide range of sociodemographic factors that are likely to be associated with air conditioning usage, we were not able to directly account for its potential role in mitigating outdoor heat effects. Fifth, the validity of epigenetic clocks across genetically and environmentally diverse populations has yet to be established. As population-specific clocks are not yet widely available, we were unable to adjust our analyses for these genetic and environmental variations. Further research is needed to examine the association between outdoor heat and epigenetic aging using clocks that are validated and refined for diverse groups. Sixth, while we observe associations between heat and accelerated epigenetic aging, it is important to acknowledge that epigenetic modifications may represent adaptive responses to heat through acclimation or acclimatization, rather than solely maladaptive changes associated with accelerated aging. The current study's observational design does not allow us to definitively distinguish between these adaptive and maladaptive processes. Nor do we identify specific CpG sites associated with increased ambient heat in this study. Future research is needed on how methylation changes with heat in order to provide insights into normal physiological processes and aging-related epigenetic alterations that occur in response to heat exposure.

Appendix S2. Heat Index Equations

The following base equation and adjustments are used to calculate the heat index using the gridMet data are given below.

Base Equation
When HI ≥ 80 °F (26.7 °C),

$$HI = -42.379 + 2.04902523\,T + 10.14333127\,RH - 0.22475541\,T\,RH - 0.00683783\,T^2 - 0.05481717\,RH^2 + 0.00122874\,T^2RH + 0.00085282\,T\,RH^2 - 0.00000199\,T^2RH^2$$

Where T is temperature °F and RH is relative humidity (%).

When HI < 80 °F (26.7 °C), use the following simplified equation in lieu of the base formula:

$$HI = 0.5\,(T + 61.0 + [\,(T - 68.0) * 1.2] + 0.094\,RH)$$

Adjustment Equation
We apply the following adjustments to the heat index formula, based on the work by Dahl et al (*34*).

Adjustment 1:
When 80 °F (26.7 °C) ≤ T ≤ 112 °F (44.4 °C) and RH < 13%, subtract the following adjustment HI_{adj1} from the calculated HI:

$$HI_{adj1} = \left(\frac{13 - RH}{4}\right) * \sqrt{\frac{17 - \text{abs}(T - 95)}{17}}$$

Adjustment 2:
When 80 °F (26.7 °C) ≤ T ≤ 87 °F (30.6 °C) and RH > 85%, add the following adjustment HI_{adj2} from the calculated HI:

$$HI_{adj2} = \left(\frac{RH - 85}{10}\right) * \left(\frac{87 - T}{5}\right)$$

Appendix S3. Model Specification

$$\text{Epigenetic Clock}_{\text{ij}} = \gamma_{00} + \gamma_{01}\text{Heat}_{\text{j}} + \beta_{\text{yx}}\text{X}_{\text{ij}} + \beta_{\text{yz}}\text{Z}_{\text{j}} + u_{0j} + r_{ij}$$

where subscripts j represents census tract (level 2) and i represents individual (level 1).

$\text{Epigenetic Clock}_{\text{ij}}$ is the value of epigenetic aging measures (i.e., PCHorvathAge acceleration, PCHannumAge acceleration, PCPhenoAge acceleration, PCGrimAge acceleration, or DunedinPACE) for individual i at census tract j. γ_{00} is the overall intercept (i.e., the average value of the epigenetic clock when all predictors are zero). γ_{01} represents the effects of ambient outdoor heat (Heat_{j}) on the epigenetic clock. X_{ij} is a vector of individual-level covariates (i.e., cell-type composition, age, gender, race/ethnicity, education in years, household wealth, smoking, alcohol use, obesity, and physical activity) for the individual i in census tract j. Z_{i} is a vector of tract-level covariates (tract social vulnerability scores, urbanicity, O_3, and $PM_{2.5}$) for census tract j. β_{yx} and β_{yz} denote vectors of coefficients for covariates X_{ij} and Z_{i}, respectively. $u_{0\text{j}}$ is a random intercept of census tract j. r_{ij} is the individual-specific error term (residual).

Fig. S1. Subgroup Analyses in the Association between Ambient Outdoor Heat and Accelerated Epigenetic Aging

A. PCPhenoAge Acceleration

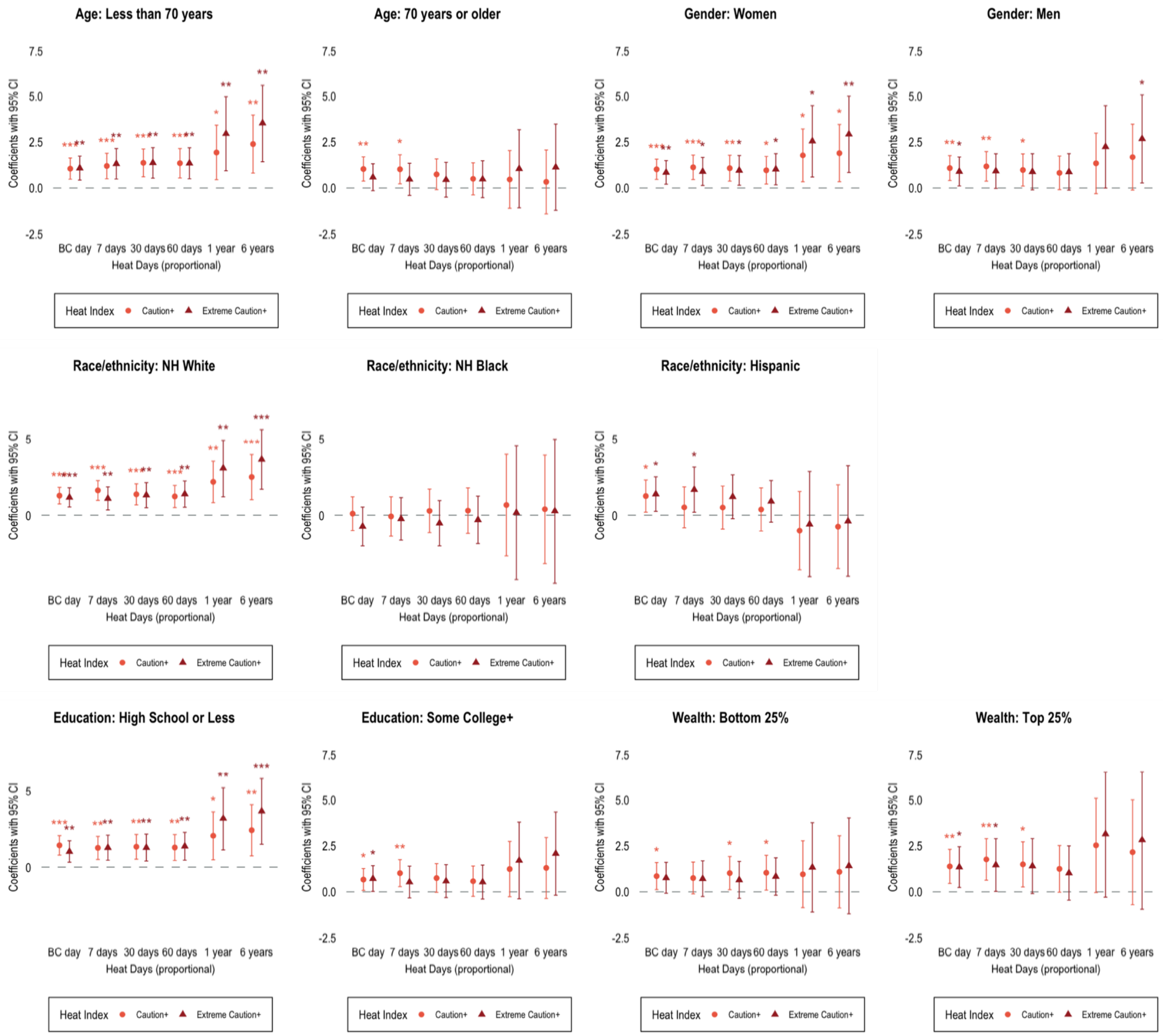

B. PCGrimAge Acceleration

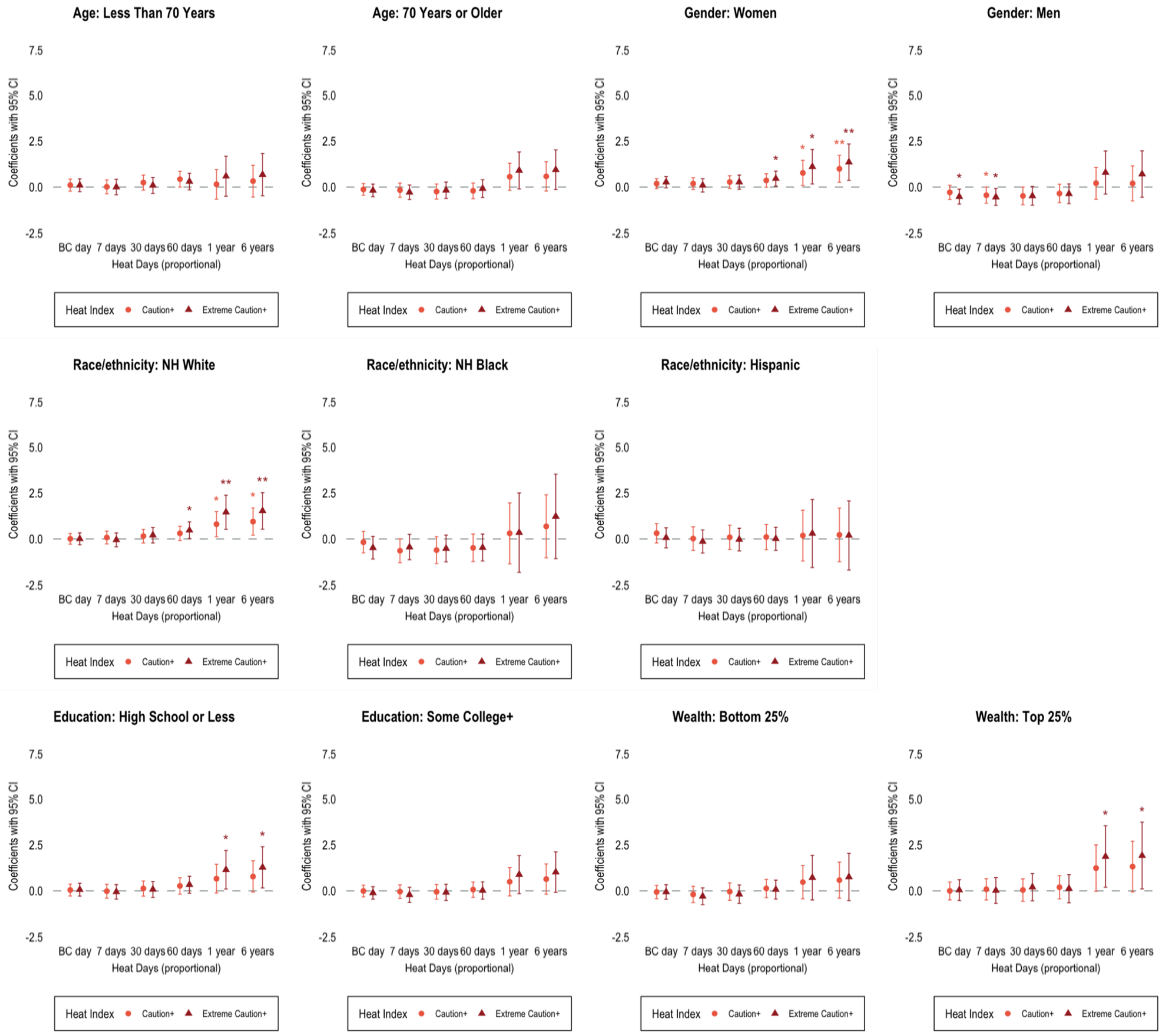

C. DunedinPACE

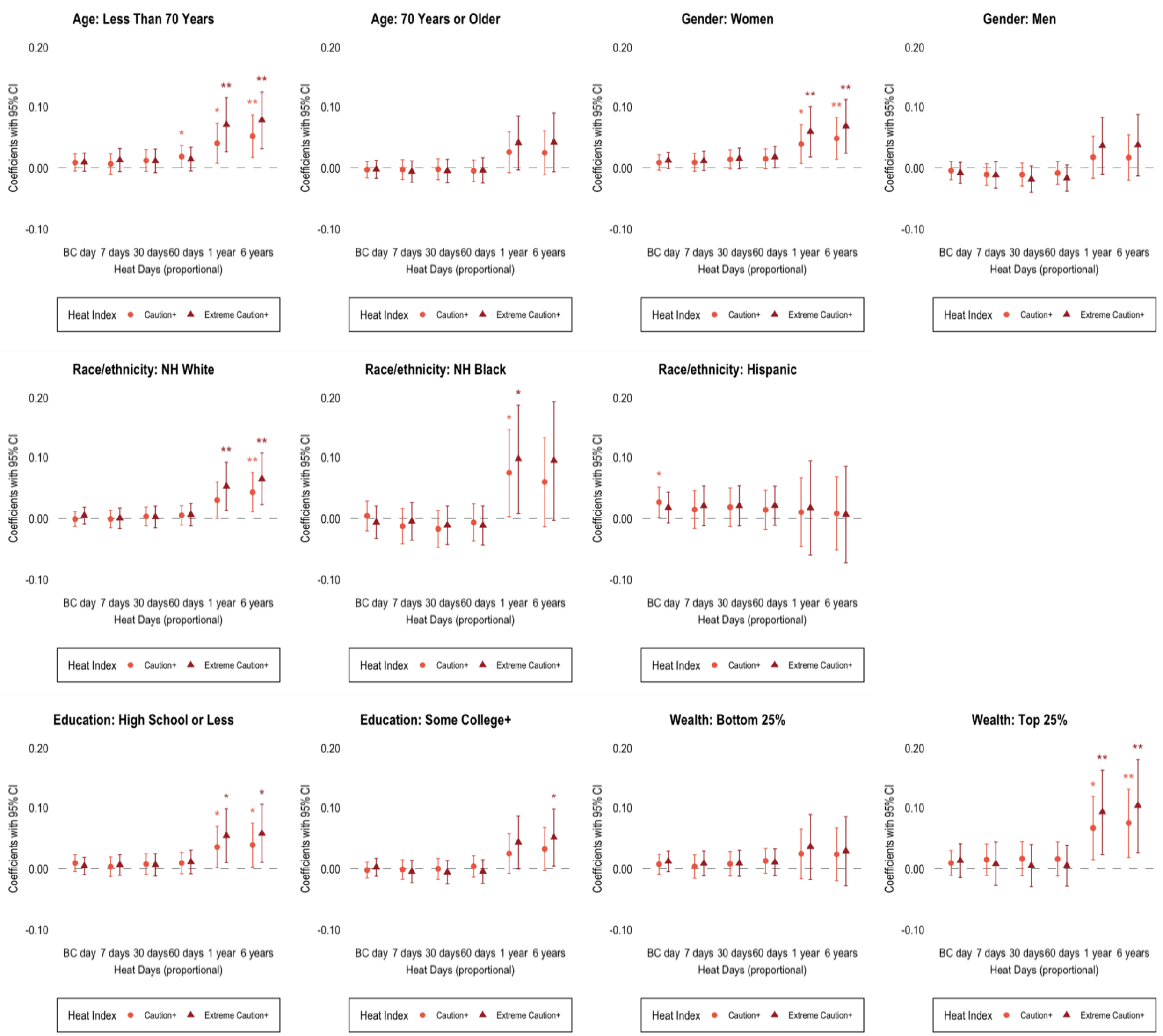

Note. * $p < .05$; ** $p < .01$; *** $p < .001$. B represents coefficient for heat, derived from separate models where each epigenetic clock is regressed on each heat measure across two heat levels and six windows. p values are two-tailed and test the null hypothesis that the estimated B is equal to 0, based on t-statistics.

Stratified models are estimated for each subgroup. All models are adjusted for confounders that are potentially associated both outdoor heat and epigenetic clocks: cell types (i.e., %monocyte, %NK, %B, %CD8, %CD4), age, sex, race/ethnicity, education, household wealth, smoking status, drinking status, obesity, physical activity, tract-level social vulnerability, urbanicity, and mean levels of O_3 and $PM_{2.5}$ for the same time windows used for outdoor heat measures. Ambient outdoor heat is measured by calculating the total number of heat days within each time window (i.e., the day of the blood collection, prior 7 days, 30 days, 60 days, 1 year, and 6 years before the blood collection date). To facilitate comparison across different time windows, the proportion of number of heat days, rather than the total count, is used.

Fig. S2. Sample Distribution by US Census Divisions

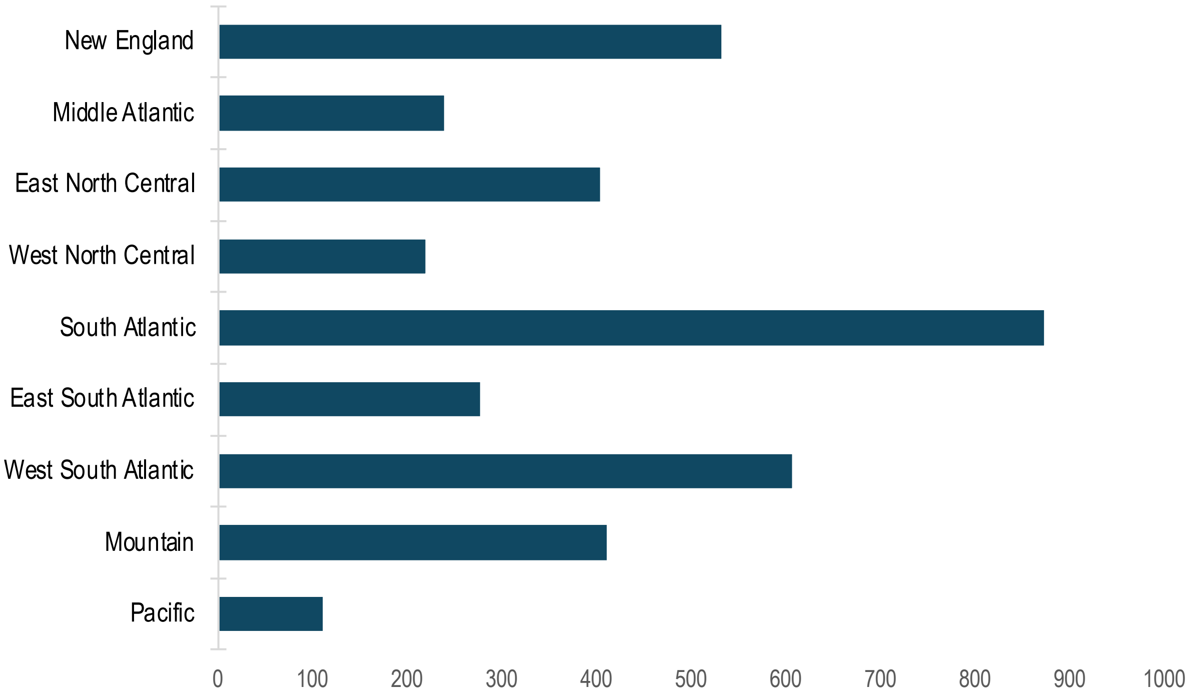

	Pacific	Mountain	West South Atlantic	East South Atlantic	South Atlantic	West North Central	East North Central	Middle Atlantic	New England
%	14%	7%	11%	6%	24%	8%	17%	11%	3%
■ N	533	240	406	221	875	278	609	412	112

Note. N shows the raw counts and % is weighted using the survey weights.

Fig. S3. Sample Selection

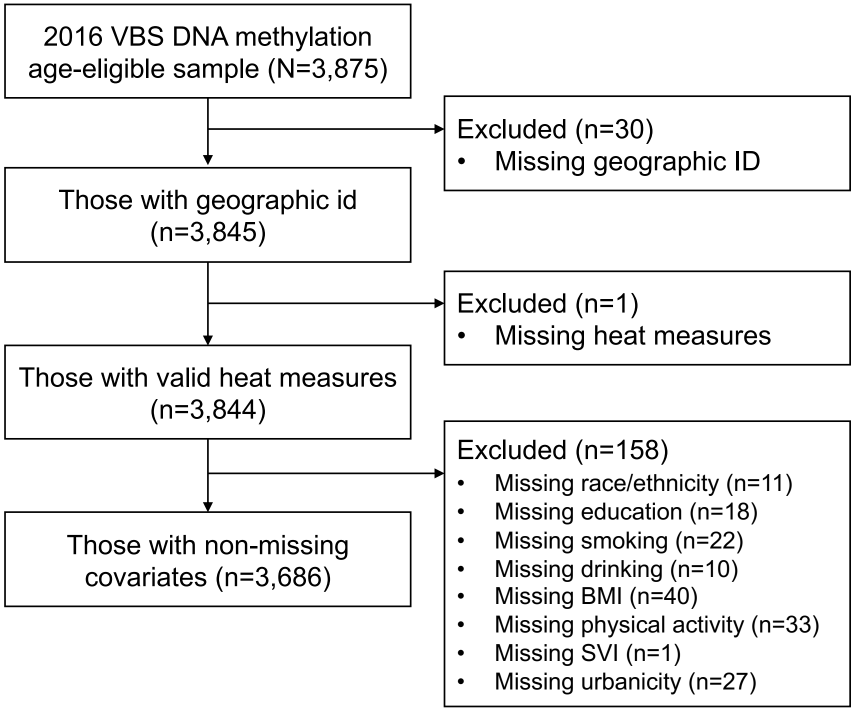

Note. For analysis of 6-year heat exposure, additional 7 respondents are excluded due to missing or incomplete geographic information over time, which results in the final analytic sample of 3,679.

Fig. S4. Distributions of Epigenetic Clocks

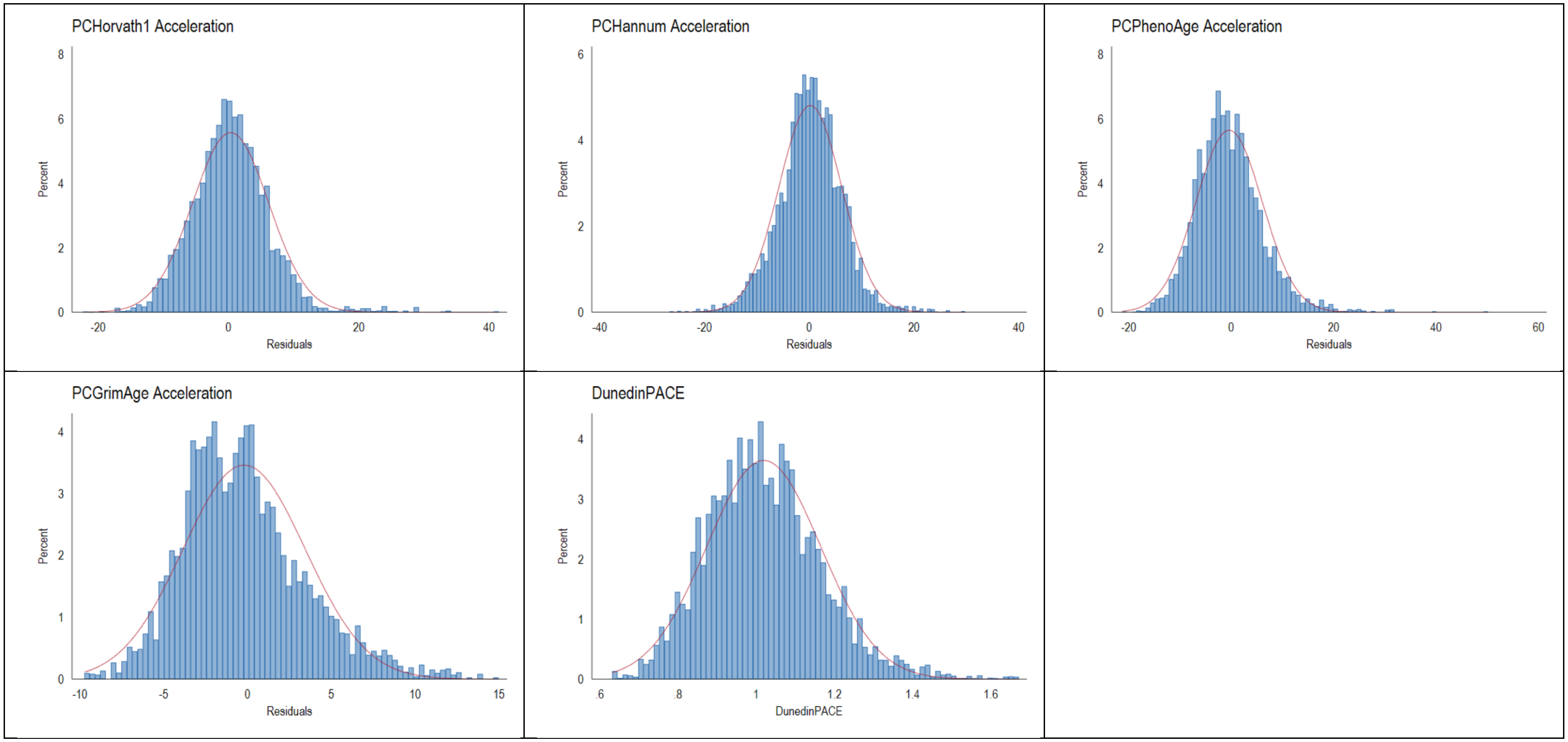

Fig. S5. Patterns of Missing Data in Cell-types Composition

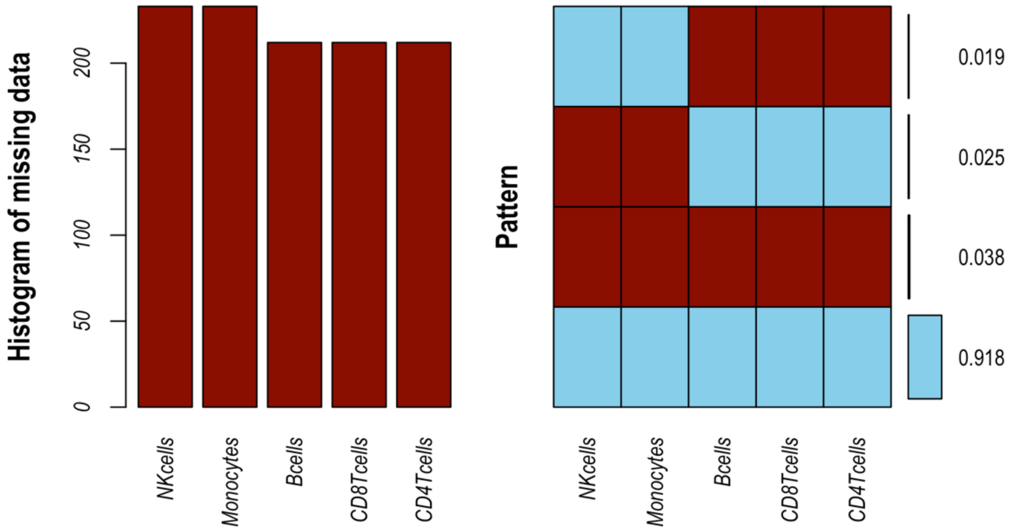

Note. This figure displays the missing data aggregation plot. On the left, the histogram represents the distribution of missing data, with the number of missing observations for each variable. On the right, the missing pattern analysis (created using the r VIM package) visualizes the different patterns of missing data across variables. The percentages displayed on the right side represent the proportion of the dataset that follows each missing pattern. 91.8% of our sample have no missing values for any of the measured cell types.

Table S1. Differences in Ambient Outdoor Heat Days Across Time Windows by Covariates

	Caution+						Extreme Caution+					
	BC	7 days	30 days	60 days	1 year	6 years	BC	7 days	30 days	60 days	1 year	6 years
Variables	%	M ± SD	M ± SD	M ± SD	M ± SD	M ± SD	%	M ± SD	M ± SD	M ± SD	M ± SD	M ± SD
Age												
Less than 70	39%	3.1 ± 3.4	12.1 ± 12.3	24.8 ± 23.1	125.4 ± 67.2	728.2 ± 373.8	17%	1.3 ± 2.5	5.5 ± 9.2	11.4 ± 17.9	53.1 ± 50.2	305.5 ± 278.9
70+	48%	3.7 ± 3.4	14.0 ± 12.0	27.0 ± 22.3	129.0 ± 70.0	761.8 ± 390.3	21%	1.7 ± 2.8	6.4 ± 9.8	12.1 ± 18.1	54.9 ± 52.1	328.9 ± 290.5
p-value	<0.001	<0.001	0.009	0.106	0.382	0.148	0.041	0.01	0.1	0.491	0.578	0.225
Gender												
Men	40%	3.1 ± 3.3	12.3 ± 12.1	24.9 ± 22.8	126.6 ± 68.6	735.0 ± 378.1	16%	1.3 ± 2.5	5.3 ± 9.0	10.9 ± 17.3	53.5 ± 51.1	308.7 ± 281.6
Women	45%	3.5 ± 3.4	13.4 ± 12.3	26.4 ± 22.8	127.0 ± 68.0	746.8 ± 382.9	21%	1.6 ± 2.7	6.3 ± 9.9	12.3 ± 18.6	54.0 ± 50.9	319.7 ± 285.5
p-value	0.004	0.024	0.066	0.156	0.908	0.504	0.018	0.002	0.012	0.082	0.835	0.38
Race/ethnicity												
Non-Hispanic White	41%	3.2 ± 3.3	12.3 ± 12.0	24.6 ± 22.4	118.8 ± 63.9	694.9 ± 356.0	17%	1.4 ± 2.5	5.3 ± 8.9	10.5 ± 16.8	47.8 ± 47.2	281.7 ± 261.8
Non-Hispanic Black	51%	3.8 ± 3.4	14.9 ± 12.3	29.8 ± 22.7	142.4 ± 58.2	839.0 ± 323.9	20%	1.7 ± 2.8	7.3 ± 10.4	14.7 ± 19.6	68.3 ± 45.4	393.4 ± 246.3
Hispanic	50%	4.1 ± 3.6	15.7 ± 13.0	31.6 ± 24.9	179.3 ± 86.7	1038.1 ± 479.3	30%	2.2 ± 3.1	9.2 ± 12.0	19.0 ± 23.7	89.9 ± 68.9	520.5 ± 390.2
Non-Hispanic Other	42%	3.2 ± 3.5	12.1 ± 12.8	23.6 ± 23.3	132.1 ± 72.6	767.1 ± 405.3	20%	1.6 ± 2.5	5.9 ± 9.5	10.3 ± 16.9	56.3 ± 49.7	317.7 ± 285.2
p-value	0.044	0.051	0.029	0.017	<0.001	<0.001	0.045	0.089	0.047	0.03	<0.001	<0.001
Education												
High school or less	46%	3.6 ± 3.4	13.7 ± 12.3	27.3 ± 23.0	131.9 ± 69.5	773.7 ± 390.7	21%	1.7 ± 2.8	6.6 ± 10.0	13.0 ± 18.8	58.0 ± 53.0	341.8 ± 296.0
Some college	40%	3.1 ± 3.3	12.3 ± 12.1	24.5 ± 22.5	122.9 ± 67.1	716.5 ± 370.9	17%	1.3 ± 2.5	5.3 ± 9.0	10.6 ± 17.3	50.5 ± 49.1	293.6 ± 272.1
p-value	0.014	0.008	0.016	0.016	0.048	0.03	0.044	0.005	0.008	0.021	0.027	0.016
Wealth, quantile												
Q1 (Least wealthy)	45%	3.4 ± 3.5	13.5 ± 12.7	27.1 ± 23.8	142.3 ± 71.9	834.3 ± 401.4	22%	1.7 ± 2.9	7.0 ± 10.6	14.2 ± 20.3	66.2 ± 55.1	386.7 ± 309.9
Q2	45%	3.4 ± 3.4	13.4 ± 12.2	26.5 ± 22.5	126.8 ± 68.3	737.8 ± 377.4	19%	1.5 ± 2.7	6.0 ± 9.4	11.5 ± 17.4	54.0 ± 50.7	313.0 ± 278.1
Q3	43%	3.3 ± 3.3	12.5 ± 11.9	24.8 ± 22.3	119.2 ± 67.5	698.2 ± 380.3	18%	1.4 ± 2.5	5.2 ± 8.8	10.2 ± 16.6	47.5 ± 49.3	281.2 ± 278.7
Q4 (Most wealthy)	38%	3.1 ± 3.2	12.1 ± 11.8	24.1 ± 22.1	115.7 ± 60.9	675.3 ± 334.4	15%	1.2 ± 2.3	5.0 ± 8.6	10.2 ± 16.5	44.8 ± 44.3	262.4 ± 240.1
p-value	0.071	0.474	0.358	0.244	<0.001	<0.001	0.039	0.118	0.114	0.14	<0.001	<0.001
Smoking												
Never	42%	3.3 ± 3.4	12.8 ± 12.2	25.8 ± 22.8	127.5 ± 69.4	748.8 ± 389.2	19%	1.5 ± 2.6	5.9 ± 9.6	11.8 ± 18.0	54.8 ± 51.9	321.8 ± 289.8
Former	44%	3.4 ± 3.4	13.1 ± 12.1	25.7 ± 22.7	126.4 ± 67.4	735.5 ± 373.9	19%	1.5 ± 2.6	5.8 ± 9.3	11.4 ± 17.7	52.7 ± 50.2	306.9 ± 277.9
Current	40%	3.1 ± 3.4	12.2 ± 12.2	25.1 ± 23.4	125.8 ± 67.7	735.0 ± 373.4	18%	1.4 ± 2.7	5.7 ± 9.7	12.2 ± 19.0	53.9 ± 50.7	317.1 ± 282.1
p-value	0.448	0.43	0.519	0.892	0.933	0.773	0.926	0.955	0.931	0.768	0.664	0.473
Drinking												
Non-current drinker	45%	3.5 ± 3.4	13.6 ± 12.2	26.9 ± 22.8	127.7 ± 66.2	750.4 ± 373.6	21%	1.6 ± 2.7	6.2 ± 9.7	12.4 ± 18.3	54.4 ± 50.7	320.5 ± 283.7
Light drinker	40%	3.0 ± 3.3	11.9 ± 12.1	24.1 ± 22.8	126.7 ± 71.3	735.6 ± 390.4	17%	1.3 ± 2.5	5.4 ± 9.3	10.9 ± 17.8	53.7 ± 51.6	311.6 ± 285.3
Heavy drinker	36%	3.2 ± 3.3	12.4 ± 12.1	24.4 ± 22.2	120.1 ± 68.9	697.1 ± 383.2	12%	1.4 ± 2.5	5.2 ± 8.9	10.0 ± 16.5	48.4 ± 49.4	282.4 ± 274.3
p-value	0.01	0.002	0.004	0.011	0.448	0.239	0.003	0.024	0.057	0.058	0.398	0.278

Obesity												
Non-obese	43%	3.3 ± 3.4	12.9 ± 12.2	25.6 ± 22.7	125.8 ± 68.2	733.2 ± 378.7	17%	1.5 ± 2.6	5.7 ± 9.4	11.4 ± 17.8	52.7 ± 50.4	306.6 ± 279.8
Obese	43%	3.3 ± 3.4	12.9 ± 12.2	25.8 ± 22.9	128.6 ± 68.5	756.2 ± 383.9	21%	1.5 ± 2.6	6.1 ± 9.6	12.1 ± 18.3	55.6 ± 52.0	329.0 ± 290.3
Test	0.866	0.932	0.959	0.879	0.431	0.25	0.021	0.579	0.409	0.41	0.313	0.182
Physical activity												
Non-sufficient	43%	3.4 ± 3.4	13.2 ± 12.2	26.4 ± 22.8	130.7 ± 67.8	768.3 ± 380.6	20%	1.6 ± 2.7	6.2 ± 9.8	12.2 ± 18.4	57.3 ± 51.8	339.4 ± 289.3
Sufficient	42%	3.3 ± 3.4	12.7 ± 12.2	25.3 ± 22.8	124.4 ± 68.5	724.7 ± 379.9	18%	1.4 ± 2.5	5.6 ± 9.2	11.3 ± 17.8	51.6 ± 50.3	299.3 ± 279.2
Test	0.796	0.763	0.277	0.203	0.021	0.005	0.122	0.043	0.092	0.186	0.007	0.001
Social vulnerability, quantile												
Q1 (Least vulnerable)	36%	2.9 ± 3.2	11.3 ± 11.8	22.5 ± 22.0	106.5 ± 55.5	627.2 ± 311.2	13%	1.1 ± 2.2	4.5 ± 8.1	9.0 ± 15.5	38.0 ± 40.7	227.4 ± 225.6
Q2	44%	3.3 ± 3.3	12.8 ± 11.9	25.2 ± 22.2	120.7 ± 65.6	706.7 ± 368.2	19%	1.3 ± 2.5	5.2 ± 8.8	10.5 ± 16.8	49.0 ± 48.0	291.6 ± 268.9
Q3	41%	3.3 ± 3.4	12.9 ± 12.3	26.0 ± 23.1	131.3 ± 69.9	758.8 ± 383.4	19%	1.6 ± 2.7	6.1 ± 9.6	12.2 ± 18.1	56.8 ± 51.3	327.0 ± 280.2
Q4 (Most vulnerable)	50%	3.8 ± 3.5	14.6 ± 12.5	29.1 ± 23.4	149.3 ± 73.8	875.5 ± 410.9	24%	1.9 ± 2.9	7.6 ± 10.9	15.1 ± 20.6	71.5 ± 56.9	414.5 ± 320.6
Test	<0.001	0.008	0.007	0.01	<0.001	<0.001	0.004	<0.001	0.001	0.003	<0.001	<0.001
Urbanicity												
Urban	45%	3.5 ± 3.4	13.4 ± 12.2	26.6 ± 22.6	127.8 ± 67.4	747.8 ± 376.4	19%	1.6 ± 2.6	5.9 ± 9.4	11.8 ± 17.9	52.1 ± 49.1	305.5 ± 278.3
Sub-urban	48%	3.6 ± 3.4	14.4 ± 12.6	28.8 ± 23.5	143.3 ± 76.8	824.5 ± 428.9	24%	1.8 ± 2.8	7.4 ± 10.7	14.9 ± 20.4	67.4 ± 58.9	386.3 ± 330.5
Ex-urban	34%	2.7 ± 3.2	10.5 ± 11.5	21.0 ± 21.7	109.8 ± 56.8	652.9 ± 318.4	13%	1.0 ± 2.3	4.2 ± 8.2	8.4 ± 15.0	44.5 ± 43.6	266.3 ± 230.0
Test	0.021	<0.001	<0.001	<0.001	0.004	0.008	0.029	<0.001	0.001	0.002	0.042	0.054

Note. We test differences between the groups using linear regressions for continuous variables and Pearson χ^2 tests for categorical variables.

Table S2. Association between Ambient Outdoor Heat and Accelerated Epigenetic Aging

Time Window	Number of Heat Days	PCHorvathAge Accl B	LCI	HCI	PCHannumAge Accl B	LCI	HCI	PCPhenoAge Accl B	LCI	HCI	PCGrimAge Accl B	LCI	HCI	DunedinPACE B	LCI	HCI
BC day	Caution+	0.59 **	0.16	1.02	0.59 **	0.16	1.03	**1.07** ***	**0.63**	**1.51**	0.01	-0.22	0.25	0.003	-0.007	0.012
7 days	Caution+	0.55 *	0.05	1.05	0.60 *	0.10	1.11	1.15 ***	0.63	1.67	-0.04	-0.31	0.23	0.000	-0.012	0.011
30 days	Caution+	0.34	-0.19	0.86	0.24	-0.30	0.78	1.08 ***	0.53	1.62	0.03	-0.27	0.32	0.003	-0.010	0.015
60 days	Caution+	0.11	-0.43	0.66	-0.09	-0.65	0.48	0.98 ***	0.40	1.56	0.14	-0.17	0.45	0.005	-0.008	0.018
1 year	Caution+	0.43	-0.65	1.51	0.28	-0.79	1.36	1.66 **	0.56	2.77	0.62 *	0.07	1.17	0.031 *	0.007	0.055
6 years	Caution+	0.60	-0.56	1.76	0.59	-0.57	1.75	1.87 **	0.68	3.06	0.74 *	0.15	1.34	0.037 **	0.011	0.063
BC day	Extreme Caution+	0.67 **	0.19	1.15	0.64 **	0.16	1.12	0.89 ***	0.40	1.38	-0.03	-0.27	0.22	0.004	-0.007	0.014
7 days	Extreme Caution+	0.70 *	0.11	1.29	0.74 *	0.17	1.32	0.96 **	0.36	1.56	-0.10	-0.39	0.19	0.002	-0.011	0.015
30 days	Extreme Caution+	0.69 *	0.08	1.29	0.61 *	0.01	1.21	1.00 **	0.38	1.63	0.02	-0.29	0.33	0.001	-0.012	0.015
60 days	Extreme Caution+	0.50	-0.11	1.11	0.35	-0.26	0.96	1.03 **	0.38	1.67	0.18	-0.15	0.52	0.004	-0.010	0.018
1 year	Extreme Caution+	0.52	-0.91	1.95	0.32	-1.11	1.75	2.48 **	0.99	3.98	1.09 **	0.34	1.84	0.051 **	0.020	0.083
6 years	Extreme Caution+	0.92	-0.62	2.45	0.89	-0.64	2.42	2.88 ***	1.28	4.48	1.19 **	0.40	1.98	0.057 **	0.023	0.092

Note. * $p < .05$; ** $p < .01$; *** $p < .001$. Accl=Acceleration; B=Coefficient; LCI=lower 95% confidence interval; HCI=upper 95% confidence interval. B represents coefficient for heat, derived from separate models where each epigenetic clock is regressed on each heat measure across two heat levels and six windows. *p* values are two-tailed and test the null hypothesis that the estimated B is equal to 0, based on t-statistics. All models are adjusted for confounders that are potentially associated both outdoor heat and epigenetic clocks: cell types (i.e., %monocyte, %NK, %B, %CD8, %CD4), age, sex, race/ethnicity, education, household wealth, smoking status, drinking status, obesity, physical activity, tract-level social vulnerability, urbanicity, and mean levels of O_3 and $PM_{2.5}$ for the same time windows used for outdoor heat measures. Ambient outdoor heat is measured by calculating the total number of heat days within each time window (i.e., the day of the blood collection, prior 7 days, 30 days, 60 days, 1 year, and 6 years before the blood collection date). To facilitate comparison across different time windows, the proportion of number of heat days, rather than the total count, is used.

Table S3. Association between Ambient Outdoor Heat and Accelerated Epigenetic Aging, Mean Heat Index

Time	Number of	PCHorvathAge Accl			PCHannumAge Accl			PCPhenoAge Accl			PCGrimAge Accl			DunedinPACE		
Window	Heat Days	B	LCI	HCI	B	LCI	HCI	B	LCI	HCI	B	LCI	HCI	B	LCI	HCI
BC day	Caution+	0.74 **	0.22	1.25	0.72 **	0.19	1.24	0.97 ***	0.44	1.49	0.00	-0.26	0.25	0.008	-0.003	0.019
7 days	Caution+	0.93 **	0.32	1.54	0.92 **	0.32	1.52	1.03 **	0.41	1.65	-0.07	-0.37	0.22	0.004	-0.010	0.017
30 days	Caution+	0.84 **	0.21	1.47	0.73 *	0.10	1.36	1.03 **	0.37	1.68	0.00	-0.33	0.32	0.002	-0.012	0.016
60 days	Caution+	0.66 *	0.02	1.29	0.48	-0.16	1.11	1.06 **	0.38	1.73	0.14	-0.20	0.48	0.003	-0.011	0.018
1 year	Caution+	0.77	-0.62	2.15	0.48	-0.93	1.88	2.44 ***	0.99	3.89	0.99 **	0.27	1.72	0.051 **	0.020	0.082
6 years	Caution+	1.14	-0.43	2.70	1.00	-0.58	2.57	2.92 ***	1.30	4.55	1.16 **	0.35	1.97	0.062 ***	0.026	0.097
BC day	Extreme Caution+	1.16 *	0.22	2.10	1.04 *	0.14	1.94	1.56 ***	0.69	2.44	-0.34	-0.80	0.11	0.009	-0.011	0.030
7 days	Extreme Caution+	1.64 **	0.59	2.69	1.60 **	0.49	2.71	2.11 ***	1.03	3.19	-0.34	-0.86	0.17	0.008	-0.017	0.033
30 days	Extreme Caution+	1.38 *	0.26	2.50	1.31 *	0.16	2.47	1.98 ***	0.83	3.14	-0.30	-0.87	0.27	0.002	-0.024	0.028
60 days	Extreme Caution+	1.38 *	0.27	2.49	1.25 *	0.14	2.36	1.92 **	0.75	3.09	-0.06	-0.66	0.54	0.001	-0.025	0.027
1 year	Extreme Caution+	0.74	-1.94	3.42	0.14	-2.64	2.91	4.06 **	1.17	6.95	1.39	-0.04	2.82	0.083 *	0.020	0.146
6 years	Extreme Caution+	2.92	-0.55	6.39	2.25	-1.32	5.81	6.63 ***	2.92	10.35	1.91 *	0.03	3.78	0.131 **	0.050	0.213

Note. * $p < .05$; ** $p < .01$; *** $p < .001$. Accl=Acceleration; B=Coefficient; LCI=lower 95% confidence interval; HCI=upper 95% confidence interval. B represents coefficient for heat, derived from separate models where each epigenetic clock is regressed on each heat measure across two heat levels and six windows. *p* values are two-tailed and test the null hypothesis that the estimated B is equal to 0, based on t-statistics. All models are adjusted for confounders that are potentially associated both outdoor heat and epigenetic clocks: cell types (i.e., %monocyte, %NK, %B, %CD8, %CD4), age, sex, race/ethnicity, education, household wealth, smoking status, drinking status, obesity, physical activity, tract-level social vulnerability, urbanicity, and mean levels of O_3 and $PM_{2.5}$ for the same time windows used for outdoor heat measures. Ambient outdoor heat is measured by calculating the total number of heat days within each time window (i.e., the day of the blood collection, prior 7 days, 30 days, 60 days, 1 year, and 6 years before the blood collection date). To facilitate comparison across different time windows, the proportion of number of heat days, rather than the total count, is used.

Table S4. Association between Ambient Outdoor Heat and Accelerated Epigenetic Aging, Spline Models

Time	Number of Heat	PCHorvathAge Accl				PCHannumAge Accl				PCPhenoAge Accl				PCGrimAge Accl				DunedinPACE			
Window	Days Caution+	B		LCI	HCI	B		LCI	HCI	B		LCI	HCI	B		LCI	HCI	B		LCI	HCI
7 days	90th (Ref: 25th)	0.58	*	0.08	1.08	0.63	*	0.12	1.14	1.19	***	0.67	1.71	-0.03		-0.31	0.24	0.000		-0.012	0.012
30 days	90th (Ref: 25th)	0.31		-0.20	0.82	0.22		-0.30	0.74	1.01	***	0.47	1.54	0.02		-0.26	0.30	0.002		-0.010	0.014
60 days	90th (Ref: 25th)	0.12		-0.40	0.63	-0.07		-0.59	0.46	0.91	**	0.37	1.45	0.14		-0.14	0.43	0.004		-0.007	0.016
1 year	90th (Ref: 25th)	0.21		-0.35	0.77	0.12		-0.44	0.67	0.81	**	0.25	1.38	0.28		0.00	0.56	0.016	*	0.003	0.028
6 years	90th (Ref: 25th)	0.29		-0.26	0.84	0.24		-0.31	0.79	0.86	**	0.31	1.41	0.35	*	0.08	0.63	0.017	**	0.005	0.029

Note. * $p < .05$; ** $p < .01$; *** $p < .001$. Accl=Acceleration; B=Coefficient; LCI=lower 95% confidence interval; HCI=upper 95% confidence interval. B represents coefficient for heat, derived from separate models where each epigenetic clock is regressed on each heat measure across two heat levels and six windows. *p* values are two-tailed and test the null hypothesis that the estimated B is equal to 0, based on t-statistics. All models are adjusted for confounders that are potentially associated both outdoor heat and epigenetic clocks: cell types (i.e., %monocyte, %NK, %B, %CD8, %CD4), age, sex, race/ethnicity, education, household wealth, smoking status, drinking status, obesity, physical activity, tract-level social vulnerability, urbanicity, and mean levels of O_3 and $PM_{2.5}$ for the same time windows used for outdoor heat measures. Ambient outdoor heat is measured by calculating the total number of heat days within each time window (i.e., prior 7 days, 30 days, 60 days, 1 year, and 6 years before the blood collection date). To facilitate comparison across different time windows, the proportion of number of heat days, rather than the total count, is used.

Table S5. Association between Ambient Outdoor Heat and Accelerated Epigenetic Aging, Continuous Heat Index Values

Time	Heat Index	PCHorvathAge Accl			PCHannumAge Accl			PCPhenoAge Accl			PCGrimAge Accl			DunedinPACE		
Window	Values	B	LCI	HCI	B	LCI	HCI	B	LCI	HCI	B	LCI	HCI	B	LCI	HCI
BC day	Mean (std)	0.49 ***	0.26	0.72	0.53 ***	0.30	0.75	0.76 ***	0.53	1.00	0.10	-0.02	0.22	0.005 *	0.000	0.010
7 days	Mean (std)	0.37 **	0.15	0.59	0.42 ***	0.19	0.64	0.69 ***	0.45	0.92	0.07	-0.06	0.20	0.004	-0.001	0.009
30 days	Mean (std)	0.27 *	0.05	0.49	0.28 *	0.06	0.51	0.58 ***	0.35	0.81	0.08	-0.04	0.21	0.004	-0.001	0.009
60 days	Mean (std)	0.14	-0.08	0.35	0.12	-0.11	0.34	0.46 ***	0.23	0.69	0.09	-0.03	0.22	0.003	-0.002	0.008
1 year	Mean (std)	0.07	-0.14	0.28	0.08	-0.13	0.29	0.34 **	0.12	0.55	0.13 *	0.01	0.24	0.005 *	0.000	0.010
6 years	Mean (std)	0.06	-0.15	0.28	0.10	-0.12	0.31	0.32 **	0.10	0.54	0.13 *	0.02	0.24	0.005 *	0.000	0.010

Note. * $p < .05$; ** $p < .01$; *** $p < .001$. Accl=Acceleration; B=Coefficient; LCI=lower 95% confidence interval; HCI=upper 95% confidence interval. B represents coefficient for heat, derived from separate models where each epigenetic clock is regressed on each heat measure across two heat levels and six windows. *p* values are two-tailed and test the null hypothesis that the estimated B is equal to 0, based on t-statistics. All models are adjusted for confounders that are potentially associated both outdoor heat and epigenetic clocks: cell types (i.e., %monocyte, %NK, %B, %CD8, %CD4), age, sex, race/ethnicity, education, household wealth, smoking status, drinking status, obesity, physical activity, tract-level social vulnerability, urbanicity, and mean levels of O_3 and $PM_{2.5}$ for the same time windows used for outdoor heat measures. Ambient outdoor heat is measured by calculating the mean heat index values within each time window (i.e., the day of the blood collection, prior 7 days, 30 days, 60 days, 1 year, and 6 years before the blood collection date). To facilitate comparison across different time windows, standardized (std) values are used.

Table S6. Differences between Analytic Sample and Excluded Respondents with Missing

Variables	Non-Missing (N=3,686) M (SD) / %	Missing (N=189) M (SD) / %	*p*-value
Sociodemographic and health characteristic			
Age (unit: years)	68.6 (9.2)	67.5 (8.7)	0.078
Women	54.0%	54.6%	0.914
Race/ethnicity			
Non-Hispanic White	78.0%	68.9%	0.002
Non-Hispanic Black	10.0%	13.2%	
Hispanic	8.6%	16.5%	
Non-Hispanic Other	3.4%	1.3%	
Education (unit: years)	13.3 (3.0)	12.4 (4.0)	0.04
Non-housing household financial wealth [a]	6.1 (8.4)	5.8 (7.5)	0.694
Smoking			
Never	44.4%	49.3%	0.578
Former	44.6%	39.0%	
Current	11.0%	11.8%	
Drinking			
Non-current drinker	56.3%	63.7%	0.07
Light drinker	37.0%	34.5%	
Heavy drinker, 7 days a week	6.6%	1.8%	
Obesity	35.7%	38.3%	0.589
Adequate physical activity	61.8%	64.9%	0.545
Social Vulnerability Index (unit: percentile ranking)	0.5 (0.3)	0.5 (0.3)	0.139
Urbanicity			
Urban	49.9%	56.1%	0.227
Sub-urban	24.0%	27.9%	
Ex-urban	26.1%	16.0%	
Epigenetic Age			
PCHorvathAge Acceleration (unit: years)	0.4 (5.4)	-0.1 (4.9)	0.233
PCHannumAge Acceleration (unit: years)	0.3 (5.3)	-0.2 (5.2)	0.415
PCPhenoAge Acceleration (unit: years)	-0.4 (5.6)	0.2 (4.6)	0.103
PCGrimAge Acceleration (unit: years)	-0.2 (3.3)	-0.3 (2.9)	0.716
DunedinPACE (unit: rate of aging per year)	1.0 (0.1)	1.0 (0.2)	0.071

Note. [a] Non-housing household financial wealth is transformed with an inverse hyperbolic since. We test differences between the two groups using linear regressions for continuous variables and Pearson χ^2 tests for categorical variables.
